# Corporate Social Responsibility in Family Firms: Status and Future Directions of a Research Field

**DOI:** 10.1007/s10551-023-05382-4

**Published:** 2023-03-27

**Authors:** Christoph Stock, Laura Pütz, Sabrina Schell, Arndt Werner

**Affiliations:** 1 University of Siegen, Unteres Schloß 3, 57076 Siegen, Germany; 2 Institute for New Work, Bern University of Applied Sciences, Brückenstrasse 73, 3005 Bern, Switzerland

**Keywords:** Systematic Literature Review, Family Firms, Corporate Social Responsibility, Sustainable Family Business Theory, Antecedents, Outcomes

## Abstract

This systematic literature review contributes to the increasing interest regarding corporate social responsibility (CSR) in family firms—a research field that has developed considerably in the last few years. It now provides the opportunity to take a holistic view on the relationship dynamics—i.e., drivers, activities, outcomes, and contextual influences—of family firms with CSR, thus enabling a more coherent organization of current research and a sounder understanding of the phenomenon. To conceptualize the research field, we analyzed 122 peer-reviewed articles published in highly ranked journals identifying the main issues examined. The results clearly show a lack of research regarding CSR outcomes in family firms. Although considered increasingly crucial in family firm research, a study investigating family outcomes (e.g., family community status, family emotional well-being), as opposed to firm outcomes, is missing. This literature review outlines the current state of research and contributes to the actual debate on CSR in family firms by discussing how family firms can use CSR activities as strategic management tools. Moreover, our analysis shows a black box indicating how CSR links different antecedents and outcomes. The black box is significant since firms generally need to know where to allocate their scarce resources to generate the best outcomes. We identify nine research questions based on these findings, which we hope will inspire future research.

## Introduction

Family firms are the most common form of business organization in the world economy (La Porta et al., 1999; Rovelli et al., 2022). Although the relative size of the family firm sector differs from nation to nation, in most countries, at least 50% of the business population is made up of family firms, and in some countries, e.g., Brazil, Italy, USA, more than 90% (International Family Enterprise Research Academy, 2003). Many family firms have been operating successfully for generations—some for more than a century (Ahmad et al., 2020; Koiranen, 2002; Lorandini, 2015). As a result, they not only contribute enormously to global economic prosperity, are responsible for a large number of jobs (Soleimanof et al., 2018) and innovation drivers (Calabrò et al., 2019) but also shape the values and behavior of national economies (Memili et al., 2015).

Chua et al., (1999, p. 25) define a family firm as “a business governed and/or managed with the intention to shape and pursue the vision of the business held by a dominant coalition controlled by members of the same family or a small number of families in a manner that is potentially sustainable across generations of the family or families.” Reduced to its very core, a family firm forms a unity between the two subsystems, family and firm (Danes et al., 2008; Frank et al., 2017; Stafford et al., 1999), meaning that with growing overlap of both subsystems, the family and its family members become increasingly linked to the company, and vice versa (Izzo & Ciaburri, 2018; Rousseau et al., 2018).

Since the relationship between family members and the firm cannot be separated as easily as between non-family executives and the firm, the owning family influences the firm’s operations, culture, and social behavior (Chrisman et al., 2005; Daspit et al., 2021). Thus, family firm research states that most owning families have a strong interest in ensuring that their firm not only does well financially but is also perceived as valuable by society since the firm’s reputation is closely linked to that society (Giner & Ruiz, 2020; Handler, 1989; Lumpkin & Brigham, 2011; Yanez-Araque et al., 2021). Therefore, research argues that family firms conduct corporate social responsibility (CSR)—meaning that they “[…] integrate social and environmental concerns in their business operations and in their interaction with their stakeholders on a voluntary basis” (Commission of the European Communities, 2001, p. 6)—not only to build a competitive advantage by building superior stakeholder relationships (Bendell, 2022; Bingham et al., 2011; El-Kassar et al., 2018) but also to enhance the public image of the owning family which is closely related to the image of the firm (Campopiano & De Massis, 2015; Zientara, 2017). Although this also applies to other shareholder primacy relationships, Faller and zu Knyphausen-Aufseß (2018) found that CSR’s perceived value seems higher than average for family ownership.

Block and Wagner (2014a) found in an analysis of the S&P 500 that even large, publicly-listed companies with a high proportion of family ownership are more likely to adopt CSR than those with less family ownership. Even family-owned firms that are not considered responsible players, such as Walmart (Walton family) or Ford (Ford family), try to give something back to society through foundations such as the Walton Family Foundation or the Ford Foundation (Scott, 2009; Sutton, 1987)—admittedly, not necessarily through altruistic motives. The Sackler family—founders of Purdue Pharma and Mundipharma and accused of being responsible for the opioid epidemic in the USA, and cited in 1600 lawsuits—used reputation laundering and donations to museums and universities to try and redeem their name and un-tarnish their reputation (Ballantyne & Loeser, 2021). So, although family firms are not necessarily more socially responsible or even ethical than non-family firms, the research shows that if an owning family is involved in the business, compared to a non-family-owned firm, it will be more inclined to conduct CSR for reputational reasons (García-Sánchez et al., 2021; Palma et al., 2022; Seckin-Halac et al., 2021). Multinational corporation conglomerates such as those of Rupert Murdoch and the Koch brothers have caused irreparable damage to the climate movement and use CSR to attempt to green-wash their policies. Bosch, on the other hand, as a large, 100% family-owned firm, is considered a predominantly socially responsible player, especially in terms of its supplier management (Kumar & Vaz, 2017). In this context, however, it is essential to note that the many good deeds of small and medium-sized family firms making up the majority of the world’s business population stay unnoticed by the general public, as these firms tend not to publicize their good deeds (see, e.g., Déniz & Suárez, 2005; Discua Cruz, 2020; Niehm et al., 2008; Peake et al., 2017; Uhlaner et al., 2004). Given the severe social and environmental problems our world faces, it is crucial to understand what does or will motivate this group of firms to engage in CSR.

From the literature, we note that family firms implementing CSR have significantly more benefits compared to non-family firms (e.g., Antheaume et al., 2013; Niehm et al., 2008; Panwar et al., 2014) and that family firms with a higher overlap of family and firm will conduct more CSR (e.g., Kashmiri & Mahajan, 2010, 2014a; Uhlaner et al., 2004). Following this line of thought, we assume that when the family and firm subsystems overlap, the owning family will transfer family resources (e.g., financial, human, or social capital) to the firm, which they—at least partially—will invest in CSR activities such as involving themselves in environmental concerns; providing improved working conditions; supporting non-profit organizations; being involved in local community projects (Turker, 2009), thereby generating both firm and family outcomes.

Research on CSR in family firms has increased significantly over the last few years, and the related research field is growing (Faller and zu Knyphausen-Aufseß, 2018; Kuttner & Feldbauer-Durstmüller, 2018; Mariani et al., 2021; Preslmayer et al., 2018). Due to the variety of studies, the opportunity has come to synthesize the current state of research so that future research can depart from a common understanding. We therefore intent to provide a holistic view of the research field’s peculiarities offers; i.e., specific drivers (antecedents), activities, results (outcomes) and contextual factors of CSR in family firms. Such a framework would help better contextualize existing research and provide guidance for the further development of the research field. Consequently, we pose the following research questions:

1. Which antecedents drive a family firm’s corporate social responsibility?
2. Which outcomes do family firms realize by conducting corporate social responsibility?
3. Which of the family firm’s corporate social responsibility antecedents and outcomes correspond and are the resources used effectively to achieve the intended outcomes?

To study this phenomenon, we decided to examine, synthesize, and systemize the growing body of research on family firms’ CSR activities identifying the CSR antecedents and outcomes in family firms. Following Tranfield et al. (2003) systematic literature review approach, we analyzed 122 peer-reviewed research articles regarding CSR in family firms, applying our theoretical framework inspired by Stafford et al. (1999) Sustainable Family Business Theory (SFBT). The SFBT draws from the systems theory and a resource-based view assuming that the specific behavior of a family firm system emerges from the interaction of its subsystems (i.e., family and firm) and the associated resource transaction between the two. Therefore, our theoretical model builds on the existing research literature offering a critical analysis of family and firm antecedents and outcomes of CSR and guidance for future research regarding the phenomenon’s essence.

Consequently, we contribute to a better understanding of CSR in family firms. Firstly, our literature analysis reveals a pattern showing that family resources integrated into the firm through family influence increase the firm’s probability of conducting CSR activities. Most researchers have found that family firms can use CSR as a strategic tool to obtain favorable outcomes (e.g., Adomako et al., 2019; Craig & Dibrell, 2006; Lamb et al., 2017; Wu et al., 2014; Zientara, 2017) illustrating that family influence within a firm should not necessarily be seen as a liability but as a strategic asset.

Secondly, the literature shows that current research often suffers from a misalignment between empirical research and theory. The prevailing assumption is that family objectives drive family firm CSR activities (Preslmayer et al., 2018), thereby obtaining family *and* firm outcomes, whereas family firm CSR outcome-related studies only examine firm outcomes, not family and firm outcomes. Although there is much theorizing about family outcomes playing a significant role in family firm management, we could not identify any empirically-related findings.

Thirdly, current research does not identify which family firm antecedents are linked to which family firm outcomes by which CSR activities. Our literature analysis reveals that family firms can benefit greatly from CSR by generating both firm and family outcomes. It is, therefore, crucial for family firms to understand how and where to allocate their resources to achieve optimal results. To clarify CSR’s catalytic role in family firms and to enable family firms to deploy their resources for appropriate CSR activities constructively, we recommend that future research opens this black box and focuses on the particular CSR activities’ mediating effect on family firm antecedents and family firm outcomes.

The remainder of the paper is structured as follows: We discuss our literature review’s theoretical framework and describe the method used to establish the review’s article samples. We ascertain the current research status and identify subsequent lacunae. We then present an agenda for future research regarding CSR in family firms, deriving nine research questions using our SFBT-based theoretical framework. Finally, we discuss our findings and provide theoretical and practical implications based on our results.

## Theoretical Framework

The SFBT draws from the systems theory and a resource-based view assuming that the specific behavior of a family firm system emerges from the interaction of its subsystems (i.e., family and firm). This subsystem dynamic differentiates family firms from non-family firms. The family subsystem uses its resources to achieve its family-related goals, which can be subjective (e.g., emotional well-being) and objective (e.g., financial well-being). The firm subsystem also uses its resources, independent of the family, to achieve its business goals (Danes et al., 2008; Stafford et al., 1999). Both subsystems interact, enabling both firm and family to benefit from each other’s resource base.

Although both are independent systems, the subsystems overlap in family firms (Frank et al., 2017). The extent of this overlap between the family and firm systems varies: In family firms where the separation of family and firm is predominant, there is little overlap. Conversely, in family firms where the overlap is high, the extent of the interface of the family and firm subsystems is significant (Bergamaschi & Randerson, 2016). The more the two subsystems overlap, the more likely the owning family will attempt to influence the firm’s management (Astrachan et al., 2020; Chadwick & Dawson, 2018; Chua et al., 1999; Kuttner et al., 2021; Meier & Schier, 2021; Shanker & Astrachan, 1996; Sharma, 2004).

Most research is related to non-family firms (Miller & Le Breton-Miller, 2007). Non-family firms are not driven by trans-generational orientation or socioemotional wealth (SEW), which makes them more flexible since they do not have to consider emotional or generational aspects in their strategies. SEW explains the emotional needs of an owning family, such as identity, the ability to exercise family influence, and the perpetuation of the family dynasty (Gómez-Mejía et al., 2007). Non-family firms are not bound to a particular management pool and can be driven by short-run objectives, maximizing profits quarterly. Also, non-family and publicly-listed firms may have a more ‘democratic’ ownership and are more visible (Blodgett et al., 2011; Cruz et al., 2019; International Family Enterprise Research Academy, 2003; Miller & Le Breton-Miller, 2007).

Most researchers have found that where CSR is concerned, family firms behave differently to non-family firms (Cabeza-García et al., 2017; Cuadrado-Ballesteros et al., 2017; El Ghoul et al., 2016; Fehre & Weber, 2019; Izzo & Ciaburri, 2018) showing that family firms conduct more CSR activities than non-family firms (Faller and zu Knyphausen-Aufseß, 2018). The owning family provides the firm with a particular set of family resources: financial, human, or social capital to pursue its personal goals within the firm (Pütz et al., 2022; Weismeier-Sammer et al., 2013). *Familiness* describes the family resources integrated within the firm and is “the unique bundle of resources a particular firm has because of the system interaction between the family, its individual members, and the business.” (Habbershon & Williams, 1999, p. 11). Familiness is available regardless of the market situation (Frank et al., 2017) and can enable a family firm to overcome internal and external disruptions (Danes et al., 2008; Stafford et al., 1999).

Family firm research theorizes that family firms with significant levels of familiness combined with the firms’ resources will be able to achieve increased performance levels (Chrisman et al., 2005; Pütz et al., 2022; Weismeier-Sammer et al., 2013). The research literature shows that family firms have higher CSR levels (e.g., Dyer & Whetten, 2006; Liu et al., 2017; Singal, 2014), and we propose that this phenomenon is mainly rooted in the familiness effect where family resources (i.e., financial, human, and social capital) are provided by the owning family (Danes et al., 2008; Weismeier-Sammer et al., 2013) for the benefit of the firm and the family. The increased CSR levels are explained through the image spillover effect. Owning families are often entrenched in their local communities and set great store by their public image. CSR is a valued tool, particularly by family-owned firms (Faller and zu Knyphausen-Aufseß, 2018), that facilitates image enhancement, thereby encouraging client loyalty and embedding the firm’s reputation, consequently leading to increased financial success (Giner & Ruiz, 2020; Handler, 1989; Lumpkin & Brigham, 2011; Yanez-Araque et al., 2021).

**Fig. 1:**
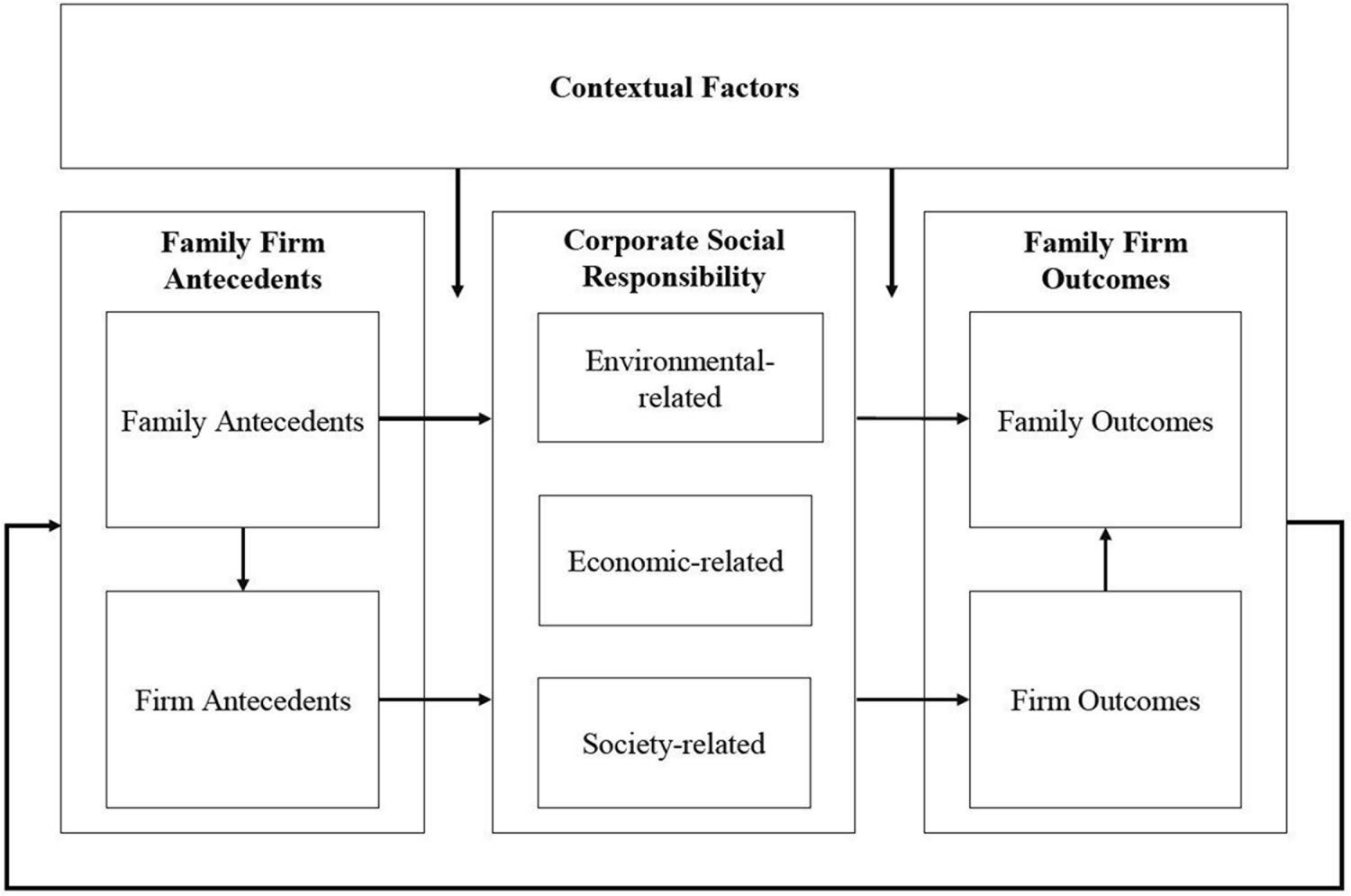
Theoretical Model

Family firms are usually trans-generationally oriented and, therefore, strive to preserve family or firm outcomes so the next generation may benefit (Bammens & Hünermund, 2020; Memili et al., 2020; Pan et al., 2018). By applying the SFBT (Stafford et al., 1999) to our research questions, we created a conceptual framework (see Fig. 1 below) dividing family firm CSR antecedents and outcomes into either the family or the firm subsystem. While family antecedents emerge from the family subsystem, firm antecedents emerge from the firm subsystem. In terms of outcomes, the family subsystem profits from the family outcomes, while the firm subsystem profits from firm outcomes. The more the two subsystems overlap, the greater the interdependencies between family and firm antecedents or outcomes. We also include a loopback from the family firm outcomes to the family firm antecedents since we believe that the outcomes from today can be the antecedents of tomorrow.

Following Elkington’s (1998) triple-bottom-line approach that states sustainable development must take place on a different dimension, we classify the applied CSR measures into environmental-, economic-, and societal-related CSR activities and postulate that the family resources provided to the firm should lead to higher levels of CSR. The family subsystem should also benefit through family outcomes. Environmental-related CSR activities are those that aim to reduce or compensate for environmentally harmful behavior, e.g., by fostering ecologically sustainable innovations, adapting green investment strategies, or adopting their behavior according to eco-certification standards (e.g., Bammens & Hünermund, 2020; Dou et al., 2019; Miroshnychenko et al., 2022; Richards et al., 2017). Economic-related CSR activities favor stakeholders who directly relate to the company’s value creation, e.g., employees, customers, or suppliers (e.g., Bennedsen et al., 2019; Dangelico, 2017; Graafland, 2020; Uhlaner et al., 2004; Zheng et al., 2017). Societal-related CSR includes generalized activities such as donations, attention to pressing community issues, or non-profit organization support (e.g., Bhatnagar et al., 2020; Bingham et al., 2011; Uhlaner et al., 2004).

CSR is a firm-level construct. Through these activities, firms maintain or strengthen relationships with different stakeholders, thereby increasing their competitive position (Stock et al., 2022; Wagner, 2010). However, due to the interdependence between family and firm, in the case of family-owned firms, both subsystems benefit from the positive effects CSR generates.

The SFBT also theorizes that a family firm is more resilient to external disruptions due to the buffer that family resources can provide (Stafford et al., 1999). Researchers must also look at moderating contextual factors outside the family firm system to understand the heterogeneous findings better. For example, when CSR is essential for stakeholders (e.g., valued by customers, society, or industry regulations), the family firm may be more incentivized to invest its resources in CSR activities (e.g., Baù et al., 2021; Chen & Liu, 2022; Samara et al., 2018). In CSR research, such factors are often understood as directly affecting CSR activities (Aguinis & Glavas, 2012). Our SFBT-based theoretical model includes contextual factors as exogenous drivers, while family and firm antecedents form endogenous drivers. In a family firm-specific context, we consider that more or fewer resources from the family or firm subsystem are allocated to CSR activities due to the pressure emerging from the context in which the family firm operates.

## Methodology

To answer our research questions, we applied the Tranfield et al. (2003) methodology, which uses three phases (i.e., planning, conducting, and reporting) to systematically review and collect significant scientific contributions in a specific research area. We developed a detailed search strategy and search protocol for English articles in peer-reviewed scientific journals. We then carried out the pre-defined search in the following databases: (1) EBSCO Business Source Elite; (2) Elsevier Science Direct; (3) Emerald; (4) Springer Link; (5) Wiley Online Library; and (6) ISI Web of Science. We searched these databases using a combination (AND conjunction) of two keyword groups. Due to the nascent stage of CSR in family firm research (Kuttner et al., 2021) and the wide range of synonyms regarding CSR (Dahlsrud, 2008; Matten & Moon, 2008; Van Marrewijk, 2013), we decided to apply a wide range of keywords. The first group dealt with the identification of CSR-relevant research using: (CSR OR ‘corporate social responsibility’ OR ‘social responsibility’ OR ‘corporate responsibility’ OR ‘corporate social’ OR ‘corporate citizenship’ OR ‘environmental management’ OR ‘sustainab*’ OR ‘social management’ OR ‘ethic’ OR SDG OR ‘sustainable development goals’). The second group concentrated on the relevant literature concerning family firms: (‘family firm*’ OR ‘family business*’ OR ‘family enterprise*’ OR ‘family sme’ OR ‘family own*’ OR ‘family-own*’ OR ‘family control*’ OR ‘family led’ OR ‘family involve*’ OR ‘family influence*’).

By screening all search results that included both a keyword from the CSR and the family firm keyword group in the title or abstract (current analysis covers published research up to June 30th, 2022), we identified 368 studies. We did not consider articles that included one term of both keyword groups but did not deal with both categories explicitly or implicitly, as was the case with studies dealing with CSR (or one of its synonyms) using family firms for the analysis without addressing their particularities. Studies dealing with the ethical values in family firms, but not their impact on CSR activities or related concepts, were also excluded. After this initial screening, we excluded all articles in journals that were not ranked as ‘2 or better’ by the Association of Business Schools’ (2021) *Academic Journal Guide*. By doing this, 122 articles remained a final sample for further in-depth analysis. Two authors read all papers independently and extracted information regarding author(s), year, title, journal, research method, applied theory, geographic scope, and critical variables using a data-extraction sheet. To better understand the articles within our sample, we also looked up the number of citations per paper using google.scholar.

The 122 articles were then categorized by whether the key variables analyzed were CSR antecedents or outcomes of family firms or both CSR antecedents and outcomes. Articles that examined the effect of family firm-specifics on CSR were classified into ‘antecedents,’ while articles classified into the ‘outcome’ category examined how family firm-specifics affect CSR’s effects. First, we subdivided antecedents and outcomes into a family and a firm’s subcategories, as suggested by the SFBT. The SFBT indicates that integrating family and firm resources helps encounter internal and external disruptions (Danes et al., 2008; Stafford et al., 1999). Consequently, we created a subcategory with contextual factors affecting a firm’s longevity, including stakeholder pressure and community embeddedness. Two authors discussed and iteratively organized all subdivisions during the analysis process. Two subsequent authors were consulted when a disagreement occurred, and all authors discussed categorization extensively until a consensus was found.

## Current Research Status

### Article Characteristics

The 122 reviewed articles were published in 52 journals, mainly on general management, ethics, gender, and social responsibility. It is noteworthy that the journal with the most significant number of publications is the *Journal of Business Ethics*, which is responsible for 18.85% of all publications in our review. We identified 15 journals, each publishing at least two articles relevant to our research field. These 15 journals account for 51.64% of all reviewed articles. The remaining 36 journals published one article each, accounting for 29.51% of all reviewed articles. Our citation analysis shows similar results. First, the 122 articles have a general citation count of 15,952. Once again, the *Journal of Business Ethics* stands out, covering 20.44% of the citations, followed by *Entrepreneurship Theory and Practice* with 18.81% and *Family Business Review* with 13.10%. Drawing on the *Academic Journal Guid*e (Association of Business Schools, 2021) to evaluate the journal quality (‘4’ being the highest score and ‘2’ the lowest), 12.30% of the reviewed articles appeared in journals ranked as ‘4’, 57.38% were ranked as ‘3’, and 30.32% ranked as ‘2’ (see Table 1).

**Table 1:** Most Influential Journals

No.	Journal Title	AJG Ranking	Number of Publications	Number of Citations
1	Journal of Business Ethics	3	23 (18.85%)	3261 (20.44%)
2	Business Strategy and the Environment	3	13 (10.66%)	803 (5.03%)
3	Family Business Review	3	10 (8.20%)	2089 (13.10%)
4	Journal of Family Business Strategy	2	6 (4.92%)	451 (2.83%)
5	Journal of Cleaner Production	2	6 (4,92%)	210 (1.32%)
6	Entrepreneurship Theory and Practice	4	5 (4.10%)	3001 (18.81%)
7	Journal of Business Research	3	3 (2.46%)	326 (2.04%)
8	Asia Pacific Journal of Management	3	3 (2.46%)	152 (0.95%)
9	Journal of Small Business and Enterprise Development	2	3 (2.46%)	405 (2.54%)
10	International Journal of Research in Marketing	4	2 (1.64%)	247 (1.55%)
Total			74 (60.66%)	10945 (68.61%)

**Fig. 2:**
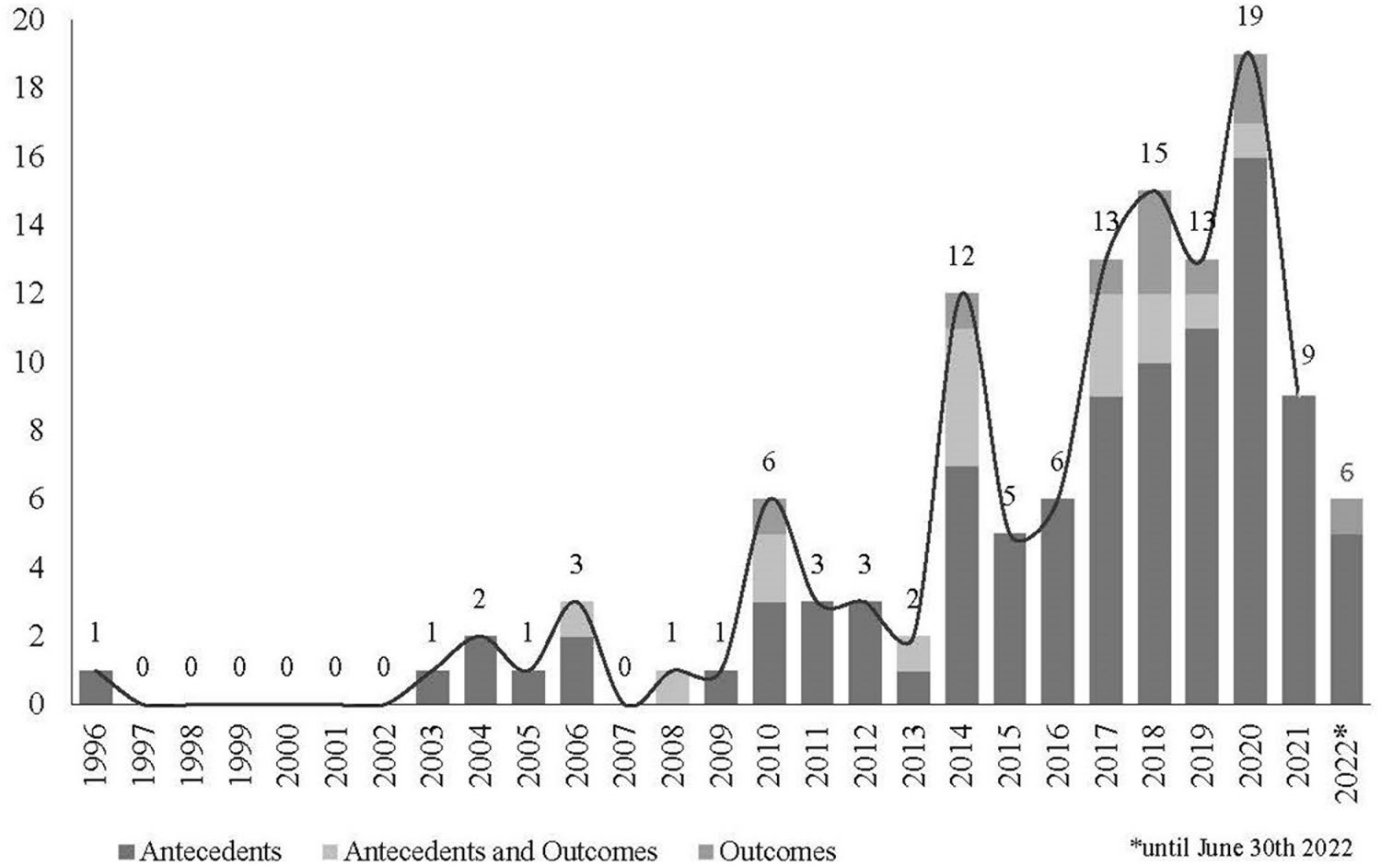
Annual Distribution of the 122 Reviewed Published Articles

The density of publications on CSR in family firms has increased significantly in the last ten years (see Fig. 2). One reason might be that CSR research, in general, became more attractive since the global financial crisis of 2007/2008, when corporate entities’ mismanagement and irresponsible behavior were discovered and made public (Blodgett et al., 2011). This crisis necessitated a major social reassessment and overhaul of business practices in financial and corporate institutions (Crane et al., 2013). Due to their trans-generational orientation (Giner & Ruiz, 2020; Lumpkin & Brigham, 2011), family firms have been discussed as a counter-model to opportunistic, shareholder-value-oriented, non-family firm management (Blodgett et al., 2011), which could explain increased research activities regarding CSR antecedents in family firms. Although it is still a significantly under-researched area, the debate about CSR’s family firm outcomes has become more popular (Kuttner & Feldbauer-Durstmüller, 2018). The relatively lower research output since 2021 could be explained by focusing on the COVID-19 pandemic. Nevertheless, given the significant social and environmental problems our world faces, our research field’s growth will likely continue (see, e.g., Le Breton-Miller & Miller, 2022). Researchers want to examine how family firms differ from non-family firms (Adams et al., 1996; Campopiano & De Massis, 2015; Maung et al., 2020) and what both types of firms can learn from these differences (Craig & Dibrell, 2006; Kashmiri & Mahajan, 2014b; Samara & Arenas, 2017).

When looking at reviewed article’s research methods (see Table 2), quantitative research stands out. When analyzing large firms, quantitative research mainly draws from longitudinal databases such as the Thomson Reuters databases (e.g., El Ghoul et al., 2016; Martínez-Ferrero et al., 2017, 2018), KLD data (e.g., Block & Wagner, 2014a, 2014b; Kim et al., 2017; Lamb & Butler, 2018; Liu et al., 2017), annual reports (e.g., Biswas et al., 2019; Sundarasen et al., 2016; Zamir & Saeed, 2020) and S&P 500 firms (e.g., Cui et al., 2018; Kashmiri & Mahajan, 2014b; Wagner, 2010). Quantitative studies examining family-owned small and medium-sized enterprises (SMEs) draw from cross-sectional surveyed data (e.g., Dawson et al., 2020; Peake et al., 2017) as there is little publicly available data on SMEs (Miller & Le Breton-Miller, 2007). Qualitative methods were used for inductive exploration of new research issues and theories, e.g., semi-structured interviews using case study methodology (see Aragón-Amonarriz et al., 2019; Bhatnagar et al., 2020; Marques et al., 2014).

**Table 2:** Research Method Used

	Antecedent-related	Antecedent- and Outcome-related	Outcome-related	Total
Quantitative	81 (66.39%)	14 (11.48%)	9 (7.38%)	104 (85.25%)
Qualitative	10 (8.20%)	1 (0.82%)	0 (0.00%)	11 (9.02%)
Conceptual	5 (4.10%)	1 (0.82%)	1 (0.82%)	7 (5.74%)
Total	96 (78.69%)	16 (13.11%)	10 (8.20%)	122 (100.00%)

### Theories in Use

In sum, we found 96 different applied theories, giving the impression that the research field’s theoretical foundation is fragmented. However, most theories played only a minor role within our sample. When analyzing the applied theories’ underlying assumptions, we noted four theories appearing at least once in 52 papers: The Principal Agency Theory, SEW, Stakeholder Theory, and Institutional Theory (see Table 3). 30 studies combine those by drawing from different assumptions to explain a family firm’s CSR activities.

**Table 3:** Theories

Theory	Representative Studies
Principal Agency Theory	Abeysekera & Fernando (2020), Block (2010), Cui et al. (2018), El Ghoul et al. (2016), Labelle et al. (2018), Seckin-Halac et al. (2021), Wiklund (2006)
Socioemotional Wealth	Cruz et al. (2014), Dick et al. (2021), Graafland (2020), Lamb & Butler (2018), Samara et al. (2018), Terlaak et al. (2018), Zientara (2017)
Stakeholder Theory	Ahmad et al. (2020), Bendell (2022), Bingham et al. (2011), Maggioni & Santangelo (2017), Delmas & Gergaud (2014), Uhlaner et al. (2004)
Institutional Theory	Agostino & Ruberto (2021), Bammens & Hünermund (2020), Campopiano & De Massis (2015), Du et al. (2016), Ge & Micelotta (2019), Kim et al. (2017), Singal (2014)

N = 122 articles

In CSR-related family firm research, the Principal Agency Theory states that the stronger the control of the owning family (through ownership shares or management), the more successfully the owning family will impose its own goals on the firm (e.g., Block, 2010; López-González et al., 2019; Sahasranamam et al., 2020; Wiklund, 2006). In this regard, twenty-four articles drew on the Principal Agency Theory, focusing on conflicts in the relationship between the principal (mainly the owning family) and the agent (mainly non-family managers), characterized by information asymmetry between the two, where the agent has an information advantage against the principal. The unequal distribution of information among these groups leads to the possibility that the agent may not act in the principal’s best interest and behaves opportunistically for personal gain (Eisenhardt, 1989; Jensen & Meckling, 1976).

The Institutional Theory was referred to in eighteen articles and focused on how firms must adapt to a different environment to gain legitimacy while conducting their business (Campopiano & De Massis, 2015; Du et al., 2016; Zamir & Saeed, 2020). Since firms ostensibly adapt their behavior to the rules of the institutional norms and routines of broader society, the Institutional Theory is used to explain social behavior in different contexts (e.g., Du et al., 2016; Ge & Micelotta, 2019; Singal, 2014). Family firm-related CSR research uses this theory mainly to examine how specific antecedents affect CSR under different contextual factors (e.g., Agostino & Ruberto, 2021; Du et al., 2016; Kim et al., 2017). For example, the location of a company’s industry influences how family ownership or management affects CSR (Chen & Cheng, 2020). The same applies to cultural contexts (Samara et al., 2018).

SEW was applied in twenty-one articles and used as a theoretical concept. The first article in our sample using SEW was published in 2014, and this concept has gained popularity ever since (Swab et al., 2020). SEW focuses on the family’s affective and non-financial goals, such as strengthening the family image and maintaining control over the own firm (Gómez-Mejía et al., 2007; Labelle et al., 2018; Marques et al., 2014; Yu et al., 2015). Therefore, the most dominant argument among studies influenced by SEW is that the owning family wants to protect its family image and therefore engages in CSR to improve that image and look good to stakeholders (e.g., Dick et al., 2021; Labelle et al., 2018; Ma et al., 2022; Madden et al., 2020; Nadeem et al., 2020).

The Stakeholder Theory was used in thirteen articles and is one of the fundamental and dominant theories of general CSR research (Freeman & Dmytriyev, 2017; Freeman et al., 2010). This theory explains why family firms are stakeholder-focused and, therefore, conduct more CSR than non-family firms (Bingham et al., 2011; Delmas & Gergaud, 2014) and is attributed to the presence of an owning family. Reasons for this include, for example, inter-generational thinking and higher awareness of stakeholder pressure compared to non-family firms (Cruz et al., 2014; Delmas & Gergaud, 2014). Additionally, CSR-related family firm research assumes that owner families use their firms to pursue financial and non-financial family goals and are more inclined to engage in CSR towards their stakeholders to achieve these goals (Bingham et al., 2011; Cruz et al., 2014).

There is a contemporary trend proposing a combination of the four prevailing theories (e.g., García-Sánchez et al., 2021; López-González et al., 2019; Sahasranamam et al., 2020; Seckin-Halac et al., 2021), although, notably, approximately 30% of the articles used no theories at all. However, more recent studies tend towards being theory-driven, indicating that the understanding of family firms has advanced.

### Content Findings

#### Family Firm Antecedents

In total, 96 of all articles in our sample (78.69%) dealt exclusively with the antecedent angle of CSR in family firms, while 16 (14.95%) dealt with both antecedents and outcomes simultaneously showing the predominance of antecedent-related focus of the field of CSR in family firms.

When the first articles addressing family antecedents (see Adams et al., 1996; Graafland et al., 2003; Gallo, 2004; Uhlaner et al., 2004) were published, it was implicitly theorized that family resources (i.e., family social capital toward stakeholders) are antecedents of CSR activities (Uhlaner et al., 2004). Subsequent publications applied different exercises to identify family antecedents of CSR, with family ownership being the most prominent measure (see Table 4).

**Table 4:** Family Antecedents

Family Antecedents	Effect on CSR	Representative Studies
Family-firm status	6	
	2 (33.33%) Positive	Gallo (2004), Palma et al. (2022)
	1 (16.66%) Negative	Dekker & Hasso (2016)
	3 (50.00%) Indistinct	Adams et al. (1996), Déniz & Suárez (2005), Graafland et al. (2003)
Family ownership	47	
	27 (57.45%) Positive	Bammens & Hünermund (2020), Kim et al. (2020), Sahasranamam et al. (2020)
	16 (34.04%) Negative	Abeysekera & Fernando (2020), El Ghoul et al. (2016), Ma et al. (2022)
	4 (8.51%) Indistinct	Bergamaschi & Randerson (2016), Labelle et al. (2018), Terlaak et al. (2018)
Family management	22	
	13 (59.09%) Positive	Abeysekera & Fernando (2020), López-González et al. (2019), Palma et al. (2022)
	6 (27.27%) Negative	Block & Wagner (2014a), Graafland (2020), Oh et al. (2019)
	3 (13.64%) Indistinct	Berrone et al. (2010), Cui et al. (2018), Terlaak et al. (2018)
Family ownership and management	23	
	12 (52.17%) Positive	Chen & Liu (2022), Dangelico (2017), Liu et al. (2017)
	4 (17.39%) Negative	Amann et al. (2012), Chen & Cheng (2020), Craig & Dibrell (2006)
	7 (30.44%) Indistinct	Doluca et al. (2018), Fritz et al. (2021), Kim & Lee (2018)
Socioemotional wealth	6	
	3 (50.00%) Positive	Dayan et al. (2019), Kallmuenzer et al. (2018), Kariyapperuma & Collins (2021)
	0 (0.00%) Negative	–
	3 (50.00%) Indistinct	Arena & Michelon (2018), Le Breton-Miller & Miller (2016), Zientara (2017)
Family generation	12	
	10 (83.33%) Positive	Dawson et al. (2020), Delmas & Gergaud (2014), Uhlaner et al. (2004)
	0 (0.00%) Negative	–
	2 (16.66%) Indistinct	Aragón-Amonarriz et al. (2019), Richards et al. (2017)
Family values	13	
	12 (92.31%) Positive	Aragón-Amonarriz et al. (2019), Marques et al. (2014), Sánchez-Medina & Díaz-Pichardo (2017)
	1 (7.69%) Negative	Zheng et al. (2017)
	0 (0.00%) Indistinct	–
Family firm name	4	
	4 (100.00%) Positive	Bingham et al. (2011); Kashmiri & Mahajan (2014a); Uhlaner et al. (2004)
	0 (0.00%) Negative	–
	0 (0.00%) Indistinct	–

N = 112 antecedent-related articles

The research reveals that most studies use family ownership (e.g., Bammens & Hünermund, 2020; Kim et al., 2020; Rees & Rodionova, 2015; Terlaak et al., 2018) followed by family management (e.g., Block, 2010; Cui et al., 2018; Oh et al., 2019) or a combination of both (e.g., Craig & Dibrell, 2006; Dangelico, 2017; Kim & Lee, 2018) for examining the effect of family antecedents on CSR in family firms. Although there is a moderate tendency towards a positive effect, no apparent effect on CSR activities can be found; this could be because these measures alone are insufficient to influence firm decisions, as the owning family cannot adequately influence internal decision-making processes by their mere presence (Terlaak et al., 2018; Yu et al., 2021). As suspected, the operational measures do not sufficiently reflect the overlap between family and company (Chua et al., 1999).

Although SEW covers the influence of the owning family much better than research using family ownership and management, it similarly shows heterogeneous results regarding CSR (e.g., Arena & Michelon, 2018; Dayan et al., 2019; Kariyapperuma & Collins, 2021; Zientara, 2017). Even though the probability that SEW emerges increases with the overlapping of the subsystems family and firm (Berrone et al., 2010), it does not have a resource-increasing character per se, which can be directed to conduct more CSR. Therefore, CSR only partially supports SEW-related goals, which also explains the heterogeneous results of the other studies (e.g., Cruz et al., 2014; Dick et al., 2021; Zientara, 2017). For example, SEW causes family firms to engage in CSR with external stakeholders more often than non-family firms in order to generate a positive image spillover effect for the owning family, but less to engage in CSR with internal stakeholders, as the owning family is afraid of losing control over their own company by making concessions to, e.g., employees (Cruz et al., 2014).

In line with our theoretical assumption, research using the family generation measure (e.g., Dawson et al., 2020; Delmas & Gergaud, 2014; Uhlaner et al., 2004) and family value measures (e.g., Aragón-Amonarriz et al., 2019; Marques et al., 2014; Sánchez-Medina & Díaz-Pichardo, 2017), which are better at capturing the overlap between family and firm, show predominantly positive effects on CSR. These studies argue that as the control of the owning family gets stronger on day-to-day operations, so does the opportunity to influence internal business decisions (Sharma & Sharma, 2011; Uhlaner et al., 2012). Probably the most significant overlap between family and firm is shown when the firm has the same name as the owning family and leads to an overall positive effect on CSR (e.g., Kashmiri & Mahajan, 2010, 2014a; Uhlaner et al., 2004). Thus, more sophisticated degrees of family influence, such as family generation, family values, and the family firm name, tend to be associated with a strong interrelation of family and firm subsystem.

Since a family firm consists not only of a family subsystem but also of a firm subsystem, research studies examined the influence of general firm antecedents on CSR activities in our sample (see Table 5). These studies answer how firm antecedents interact with family antecedents regarding CSR. They focus mainly on (internal) non-financial antecedents predominantly showing the effect on CSR and examine the effect of governance (e.g., Campopiano et al., 2014; El-Kassar et al., 2018; Terlaak et al., 2018) and non-family management (e.g., Martínez-Ferrero et al., 2017; Oh et al., 2019; Samara et al., 2018) on family firms’ CSR activities. With only two studies examining the effect of financial antecedents, it is apparent that further research is still needed.

**Table 5:** Firm Antecedents

Firm Antecedents	Effect on CSR	Representative Studies
Financial	2	
	2 (100.00%) Positive	Block (2010), Singal (2014)
	0 (0.00%) Negative	–
	0 (0.00%) Indistinct	–
Non-financial (internal)	28	
	24 (85.72%) Positive	Biswas et al. (2019), Martínez-Ferrero et al. (2017, 2018); Seckin-Halac et al. (2021)
	2 (7.14%) Negative	Graafland (2020), Madden et al. (2020)
	2 (7.14%) Indistinct	Kim & Lee (2018), Samara et al. (2018)
Non-financial (external)	4	
	3 (75.00%) Positive	Du (2015), Ge & Micelotta (2019), Martínez-Ferrero et al. (2018)
	0 (0.00%) Negative	–
	1 (25.00%) Indistinct	Richards et al. (2017)

N = 112 antecedent-related articles

#### Family Firm Outcomes

Ten studies within our sample (8.20%) examined the outcome side of CSR exclusively, while 16 (13.11%) dealt with both antecedents and outcomes simultaneously, showing the predominance of antecedent-related focus of CSR in family firms. Remarkably, most outcome-related studies found that family firms generate better results from CSR activities than non-family firms (see Table 6), indicating that family firms, in general, are better at utilizing CSR (e.g., Antheaume et al., 2013; Niehm et al., 2008; O’Boyle et al., 2010; Panwar et al., 2014).

**Table 6:** Firm Outcomes

Firm Outcomes	Effect of CSR	Representative Studies
Financial	17	
	10 (56.25%) Positive	Ahmad et al. (2020), Niehm et al. (2008), Pan et al. (2018)
	2 (12.50%) Negative	Choi et al. (2019), Lin et al. (2020)
	5 (31.25%) Indistinct	Dangelico (2017), Doluca et al. (2018), Liu et al. (2017)
Non-financial (internal)	7	
	6 (85.71%) Positive	Antheaume et al. (2013), Craig & Dibrell (2006), Wagner (2010)
	0 (0.00%) Negative	–
	1 (14.29%) Indistinct	Doluca et al. (2018)
Non-financial (external)	7	
	4 (57.14%) Positive	Ahmad et al. (2020), Samara & Arenas (2017), Sekerci et al. (2022)
	2 (28.57%) Negative	Hsueh (2018), Martínez-Ferrero et al. (2018)
	1 (14.29%) Indistinct	Zientara (2017)

N = 26 outcome-related articles

Most studies show how family firms improve their financial outcomes through, among others, the cost of capital (Wu et al., 2014) or return on new products (Kashmiri & Mahajan, 2014a), but mainly focus on the firm’s general performance (e.g., Adomako et al., 2019; Choi et al., 2019; Kashmiri & Mahajan, 2014b). Family firms can also improve their internal non-financial outcomes (Ahmad et al., 2020), such as longevity (Antheaume et al., 2013; Samara & Arenas, 2017) or innovation performance (Biscotti et al., 2018; Craig & Dibrell, 2006; Wagner, 2010) and in external non-financial areas such as firm reputation (Samara & Arenas, 2017; Zientara, 2017), credibility (Hsueh, 2018; Panwar et al., 2014), or customer orientation (Ahmad et al., 2020).

The research literature determines two main reasons family firms generate augmented outcomes through CSR. The signaling effect associated with family firm status prompts external non-financial outcomes (e.g., Martínez-Ferrero et al., 2018; Maung et al., 2020; Sekerci et al., 2022), and the familiness dynamic, which allows family firms to translate CSR into positive financial and internal non-financial outcomes (e.g., Craig & Dibrell, 2006; Pan et al., 2018; Wagner, 2010). The signaling effect means external stakeholders are less likely to perceive the family firm’s CSR activities as opportunistic green-washing, particularly in the case of SMEs where the owning family is evident (e.g., Ahmad et al., 2020; Dangelico, 2017; O’Boyle et al., 2010) but also with large, publicly listed family firms (e.g., Biscotti et al., 2018; Kashmiri & Mahajan, 2014a; Wu et al., 2014).

The familiness stream of literature is not concerned with whether family firms engage in more or less CSR than non-family firms but with the extent to which family firms can better translate CSR activities into positive outcomes (e.g., Wagner, 2010). Family firms have the advantage of asserting more influence on the operational management and increasing control over the firm’s subsystem (Doluca et al., 2018; Niehm et al., 2008). Both streams utilize the transgenerational aspect particular to family firms that ensures their strategies and aims have long-term focus leads them to use CSR to maximize positive outcomes for the owning family and the firm (e.g., Campopiano & De Massis, 2015; Niehm et al., 2008; Zientara, 2017).

We did not find research providing practical information on whether CSR-improved stakeholder relations affect family outcomes, even though the importance of family outcomes was referred to in the reviewed literature (e.g., Campopiano & De Massis, 2015; Niehm et al., 2008; Zientara, 2017). Déniz and Suárez (2005) note how owning families are personally affected by the relationships with stakeholders since they are inseparable from it. Furthermore, the findings of Aragón-Amonarriz et al. (2019) conclude that the owning family derives honors from socially responsible behavior and, therefore, could act as a basis for family outcome-related CSR research.

#### Contextual Factors

A fundamental assumption of studies analyzing family firms is that owning families are more sensitive to external contextual factors than other non-family owners, thus leading to a greater tendency to implement the requirements of external stakeholders (Ge & Micelotta, 2019). The owning family assigns greater importance to the firm’s image, as the family identifies with the firm (e.g., Amidjaya & Widagdo, 2020; Discua Cruz, 2020; Labelle et al., 2018; Zientara, 2017). If the firm carries the family name, the family and the firm’s reputation become inseparable, and maintaining or acquiring a good reputation is paramount (e.g., Abeysekera & Fernando, 2020; Bammens & Hünermund, 2020; Kashmiri & Mahajan, 2010; Pan et al., 2018; Uhlaner et al., 2004). Following this argumentation, an owning family will be more willing to provide resources to the family firm system for CSR activities intended for brand enhancement and expected from the firm by external stakeholders.

The research literature analysis revealed that contextual factors could be divided into general stakeholder pressure and community embeddedness. While public stakeholder pressure can be abstract communication from an anonymous group (e.g., industry) resulting in a generic response (e.g., via CSR reports) (e.g., Campopiano & De Massis, 2015), family firms with community embeddedness are more involved and use CSR to respond to the needs or requirements of the community (e.g., Niehm et al., 2008; Peake et al., 2017). Table 7 shows we found 26 studies covering general stakeholder pressure and ten explicitly covering the impact of family community embeddedness on a family firm’s CSR activities (e.g., Fitzgerald et al., 2010; Laguir et al., 2016; Peake et al., 2017).

**Table 7:** Contextual Factors

Contextual Factors	Effect on CSR	Representative Studies
Stakeholder pressure	26	
	18 (69.23%) Positive	Maggioni & Santangelo (2017), Yu et al. (2021), Zamir & Saeed (2020)
	1 (3.85%) Negative	López-González et al. (2019)
	7 (26.92%) Indistinct	Cuadrado-Ballesteros et al. (2015), Dayan et al. (2019), Le Breton-Miller & Miller (2016)
Community embeddedness	10	
	10 (100.00%) Positive	Baù et al. (2021), Dekker & Hasso (2016), Peake et al. (2017)
	0 (0.00%) Negative	–
	0 (0.00%) Indistinct	–

N = 35 articles

It is noteworthy that the relevance of public stakeholder pressure (e.g., industry norms, national culture) is more pronounced in studies analyzing large firms (e.g., Blodgett et al., 2011; Cruz et al., 2014; Cuadrado-Ballesteros et al., 2015). Studies on SMEs tend towards community embeddedness (e.g., Dekker & Hasso, 2016; Kallmuenzer et al., 2018; Peake et al., 2017). The community embeddedness perspective shifts the focus away from general stakeholder groups and examines the owning family’s interpersonal ties within the local community. The latter research argues that family-owned SMEs use CSR as a strategic tool to influence external stakeholders’ perception (i.e., local community) positively to closer relationships between them (Lamb et al., 2017; Uhlaner et al., 2012). Interestingly, all studies unanimously agree that family firms react with more CSR towards pressure from contextual factors.

Although most studies show the positive effect of external pressure on a firm to conduct CSR activities, this is country and region-dependent (Ertuna et al., 2019; Ge & Micelotta, 2019; Labelle et al., 2018; Zamir & Saeed, 2020). The first studies with US American datasets were conducted between 2003 and 2013. The economic relevance of Asia has recently increased, engendering an increase in CSR-related family firm studies and applying Asian datasets since 2009 (see Table 8). While studies using US American data mainly recorded positive effects of family antecedents on CSR (e.g., Cordeiro et al., 2020; Lamb & Butler, 2018; McGuire et al., 2012; Panwar et al., 2014), Asian studies have frequently shown the contrary (Biswas et al., 2019; El Ghoul et al., 2016; Huang et al., 2009; Muttakin & Khan, 2014).

**Table 8:** Research-originating countries

	Before 2001	2001–2005	2006–2010	2011–2015	2016–2020	Since 2021	Total
International	0 (0.00%)	1 (0.82%)	0 (0.00%)	2 (1.64%)	11 (9.02%)	6 (4.92%)	20 (16.39%)
USA	1 (0.82%)	0 (0.00%)	9 (7.38%)	11 (9.02%)	11 (9.02%)	1 (0.82%)	33 (27.05%)
Central-America	0 (0.00%)	0 (0.00%)	0 (0.00%)	0 (0.00%)	3 (2.46%)	0 (0.00%)	3 (2.46%)
Australia	0 (0.00%)	0 (0.00%)	0 (0.00%)	0 (0.00%)	1 (0.82%)	1 (0.82%)	2 (1.64%)
Europe	0 (0.00%)	3 (2.46%)	0 (0.00%)	6 (4.92%)	13 (10.66%)	4 (3.28%)	26 (21.31%)
Asia	0 (0.00%)	0 (0.00%)	1 (0.82%)	5 (4.10%)	22 (18.03%)	2 (1.64%)	30 (24.59%)
Africa	0 (0.00%)	0 (0.00%)	0 (0.00%)	0 (0.00%)	1 (0.82%)	0 (0.00%)	1 (0.82%)
Conceptual	0 (0.00%)	0 (0.00%)	1 (0.82%)	1 (0.82%)	4 (3.28%)	0 (0.00%)	7 (5.74%)
Total	1 (0.82%)	4 (3.28%)	11 (9.02%)	25 (20.49%)	66 (54.10%)	15 (12.30%)	122 (100.00%)

The reason Western cultures incorporate CSR more than their Asian counterparts can be attributed to the difference in cultural and political aims and values. Western countries tend to be highly stakeholder-oriented, and the values are based on “liberal democratic rights, justice, and societal structures” (Amann et al., 2012, p. 331), leading to more significant institutional pressure for firms to comply accordingly (Campopiano & De Massis, 2015; Dekker & Hasso, 2016). Asian countries have a more shareholder-oriented culture. Therefore, there is less social pressure to become CSR-compliant (El Ghoul et al., 2016), and the owning families tend to focus more on their personal financial well-being, subsequently regarding CSR as relatively inconsequential (e.g., Biswas et al., 2019; Du, 2015; Du et al., 2016; Muttakin & Khan, 2014). Family firms form the backbone of the Asian economy, with family ownership being the most dominant ownership form of companies in the Asia Pacific region (El Ghoul et al., 2016). Of the largest 500 largest global family firms ranked by revenue, over 20% are Asia-based, with combined revenue of almost $2 trillion (Global Family Business Index, 2021). Although it is not required by law for Asian companies to be CSR compliant, there is a trend towards encouraging more CSR from companies to entice foreign investment. Foreign investors from Western countries and companies are encouraged by their external stakeholders to provide ethically and ecologically sourced products, and therefore the investors and companies will require CSR from the Asian company they are importing from or collaborating with (Cordeiro et al., 2018; Du et al., 2018; Muttakin & Khan, 2014).

In India, for example, many local family firms are voluntarily socially responsible (Bhatnagar et al., 2020; Sahasranamam et al., 2020). They aim at better working conditions for employees, for example, and are involved in improving the local community; e.g., the Godrej Group preferred to protect mangroves on its land in Mumbai, despite the insatiable demand for housing development. Jardine Matheson, a Fortune 500 and Hong Kong-based multinational conglomerate controlled by the Keswick family has pledged its commitment to biodiversity and is implementing sustainability strategies in its many operating companies. The company has a proactive approach and collaborates with public stakeholders on climate issues, ecological sustainability, and forest protection aiming to mitigate any adverse impact from its operations and products (Jardine Matheson, 2021).

From the research literature, we conclude that it is not only essential to understand the effects of different organizational settings (i.e., family and firm subsystem) on CSR (Dahlsrud, 2008) but also the effects of external contextual and cultural factors that influence the internal processes of a family firm when considering CSR as a driver.

#### Corporate Social Responsibility Activities

We examined which CSR activities were used in our study samples and how their antecedents and outcomes differed. According to Elkington’s (1998) triple-bottom-line approach, we classified the applied CSR measures into environmental-, economic-, and societal-related CSR activities (see Table 9).

**Table 9:** Corporate Social Responsibility Activities in Family Firms

CSR Activities	Effect in Family Firms	Representative Studies
Aggregated CSR	59	
	37 (62.71%) Positive	Fitzgerald et al. (2010), Gallo (2004), Memili et al. (2020)
	12 (35.59%) Negative	Biswas et al. (2019), Hsueh (2018), Muttakin & Khan (2014)
	10 (16.95%) Indistinct	Bergamaschi & Randerson (2016), Iyer & Lulseged (2013), Zientara (2017)
Environmental-related CSR	29	
	17 (58.62%) Positive	Block & Wagner (2014b), Delmas & Gergaud (2014), Terlaak et al. (2018)
	6 (20.69%) Negative	Amann et al. (2012), Dekker & Hasso (2016), Nadeem et al. (2020)
	6 (20.69%) Indistinct	Adomako et al. (2019), Kim & Lee (2018), Doluca et al. (2018)
Economic-related CSR	21	
	14 (66.66%) Positive	Cruz et al. (2019), Kashmiri & Mahajan (2014a), López-González et al. (2019)
	4 (19.05%) Negative	Amann et al. (2012), Nadeem et al. (2020), Zheng et al. (2017)
	3 (14.29%) Indistinct	Campopiano & De Massis (2015), Cruz et al. (2014), Fritz et al. (2021)
Societal-related CSR	13	
	10 (76.92%) Positive	Bingham et al. (2011), Niehm et al. (2008), Sahasranamam et al. (2020)
	0 (0.00%) Negative	–
	3 (23.08%) Indistinct	Amann et al. (2012), Kim & Lee (2018), Block & Wagner (2014b)

N = 122 articles

Twenty-nine articles were allocated to environmental-related CSR (23.77%), 21 to economic-related CSR (17.21%), and thirteen articles to societal-related CSR (10.66%). Furthermore, we found that in 59 articles (48.36%), the majority of research is based on CSR measures that do not differentiate between different activities but average different activities in one measure (e.g., Gallo, 2004; Hsueh, 2018; Iyer & Lulseged, 2013; McGuire et al., 2012). Looking at different family and firm subsystem antecedents and outcomes through the lens of individual CSR activities gives us a balanced perspective. None of the CSR activities focus on specific antecedents or outcomes, and there are no significant differences in the effects between the activities. We conclude that the related CSR activities have not yet been sufficiently differentiated in family firm research, and the disproportionately large number of articles that do not distinguish between different CSR activities supports this view.

## Future Directions of Research

**Fig. 3:**
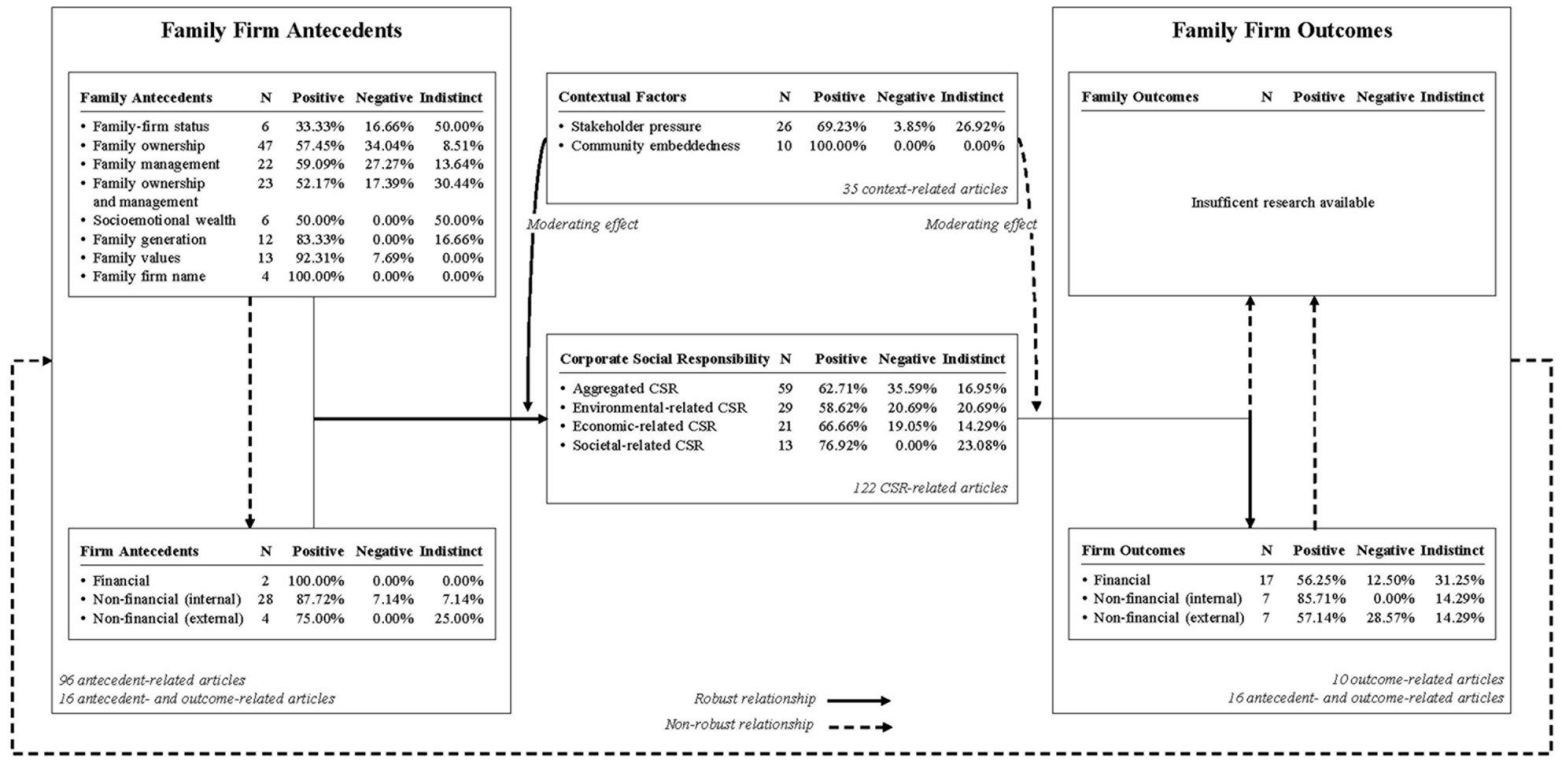
Model of Antecedents and Outcomes of CSR in Family Firms

Figure 3 recapitulates the scope and robustness of the findings in our data sample. In terms of scope, the literature shows that most CSR-related family firm research focuses on CSR antecedents, and only a few studies are concerned with outcomes. On the antecedent side, research mainly focuses on the direct effect of family antecedents on CSR or the interaction with firm antecedents and its effect on CSR. However, such research examining the interaction of family and firm antecedents on CSR is in the minority. The research clearly shows a lack of analysis on CSR outcomes in family firms, and in terms of firm outcomes, family firms achieve more through CSR than non-family firms. Although there is increasing emphasis on family firm research, the study of family outcomes (e.g., family community status, family emotional well-being) is lacking. Moreover, the catalytic role of CSR activities has not yet been studied in detail, where the fundamental question of which antecedents and outcomes are linked by which CSR activities remain unanswered.

In terms of robustness, it is evident that the more substantial the interrelation between family and firm (see, e.g., family generation, family values, and the family firm name), the higher the probability that a family firm will conduct CSR. Although the research field is mainly antecedent-oriented, the literature shows that the effect of family on firm antecedents remains unexplored. On the outcome side, CSR predominantly has robust effects on firm outcomes. Considering that an empirical examination of family outcomes is missing, it is unclear how CSR and firm outcomes affect family outcomes. Furthermore, the research literature cannot provide robust findings on how contextual factors affect the outcome side of CSR in family firms.

To gain further insight, we propose nine research questions for future exploration to open the ‘black boxes’ and consequently lead to clarifying significant aspects concerning family firm CSR activities (see Table 10).

**Table 10:** Research Questions

Research question 1a	Which firm antecedents (i.e., firm resources) affect the association between family antecedents (i.e., family resources) and CSR activities?
Research question 1b	Which conflicts can arise during the resource transaction between family and firm subsystem, and how does this affect CSR activities?
Research question 2	Which family outcomes (i.e., family resources) can an owning family generate through the CSR activities of its firm, and how do those affect the family firm’s CSR activities in subsequent periods?
Research question 3a	Which firm outcomes (i.e., firm resources) affect the association between CSR activities and family outcomes (i.e., family resources)?
Research question 3b	Which conflicts can arise during the resource transaction between family and firm subsystem, and how does this affect the family firm’s CSR outcomes?
Research question 4	Which contextual factors affect the relationship between CSR activities and outcomes (i.e., family and firm outcomes) of family firms?
Research Question 5a	Which CSR activities (e.g., environmental, economic, or societal-related) are linked to which antecedents (i.e., family and firm antecedents) and outcomes (i.e., family and firm outcomes)?
Research question 5b	How and why do CSR activities affect antecedents and outcomes of family firms?
Research question 6	How and why do CSR activities increase the longevity of family firms?

Family firm research traditionally focuses on examining family antecedents and only marginally includes firm antecedents in their models. Family and firm antecedents’ effects on CSR are mainly examined independently. Research shows that family involvement (identification and commitment) and family values have a positive effect on CSR activities but do not examine the extent to which family and firm antecedents interact with each other (e.g., Marques et al., 2014; Peake et al., 2017; Sharma & Sharma, 2011; Uhlaner et al., 2012).

Following our SFBT-based theoretical framework, we know that the higher the influence of the owning family within its firm, the greater the interaction between family and firm, and the more resources can be transferred between both (Stafford et al., 1999). When family resources are transferred to the firm subsystem, familiness is generated, providing the family firm with a more extensive resource base, ultimately leading to a competitive advantage in the long term (Frank et al., 2017; Habbershon & Williams, 1999; Weismeier-Sammer et al., 2013). These theoretical assumptions are implicitly applied, explaining that owning families involved within the firm introduces responsible behavior, which stakeholders will eventually repay (e.g., Aragón-Amonarriz et al., 2019; Fitzgerald et al., 2010; Fritz et al., 2021). According to research, the family’s social capital is a crucial driver of a family firm’s CSR activities and competitiveness (e.g., Niehm et al., 2008; Peake et al., 2017; Uhlaner et al., 2004).

Unexamined is the permeability of the two subsystem boundaries and how those affect the effectiveness of the resource transaction. Utilizing the system’s theoretical perspective, we theorize that the subsystem’s boundary permeability can differ (Frank et al., 2017). Depending on how strong the subsystem boundaries are, the impact of family antecedents (i.e., family resources) can be more or less effective on firm antecedents (i.e., firm resources). If the subsystem permeability is low, resources can easily be transferred from one subsystem to another, while such a transaction will be more difficult when the permeability of the subsystem’s boundaries is high (Danes et al., 2008; Hernes & Bakken, 2003). However, this permeability can change, e.g., if the non-family management wants to preserve power within the firm subsystem and tries to hamper the integration of family resources, meaning that the potentially positive effect of family resources (i.e., familiness) would not be achieved.

Thus, although we found that future family firm research should focus on the outcome angle of CSR, we believe that the antecedent’s research angle should also be developed. In this regard, we also propose to examine which factors could hamper the transfer of family and firm subsystem resources between the subsystems and whether this could affect the family firm’s CSR activities.

- Research Question 1a: Which firm antecedents (i.e., firm resources) affect the association between family antecedents (i.e., family resources) and CSR activities?
- Research Question 1b: Which conflicts can arise during the resource transaction between family and firm subsystem, and how does this affect CSR activities?

Family firm research mainly concentrates on examining CSR’s antecedent angle. Previous findings dealt with the financial and non-financial firm outcomes while only theorizing about family outcomes without empirically studying them. However, more and more studies have recently scrutinized CSR outcomes in family firms (e.g., Hsueh, 2018; Lin et al., 2020; Sekerci et al., 2022). The empirical scrutiny on CSR family outcomes is logical, considering CSR is a firm-level construct.

It is, however, an assumption of the SFBT-based theoretical framework that while family and firm share their resources to some extent, the family and the firm pursue their specific goals separately (Danes et al., 2008; Stafford et al., 1999). Thus, Campopiano and De Massis (2015) state that owning families can profit from the image-enhancing effect of CSR themselves through an increased family image. Furthermore, Aragón-Amonarriz et al. (2019) conclude that family honorableness is one of the outcomes of a family firm’s CSR activities, indicating that CSR generates family outcomes. However, which family outcomes can be generated through CSR has not yet been examined. Consequently, we recommend exploratory (i.e., qualitative) work in this area to determine which family outcomes an owning family may achieve through CSR.

The literature indicates that if CSR activities do not bring positive results for the owning family, they will probably cease to provide resources for CSR implementation (Palma et al., 2022). Taking a closer look at the implicit assumptions made by the reviewed studies on family outcomes (e.g., family harmony, family well-being), we find indications that a family firm’s CSR could also have an impact on the owning family itself (e.g., Campopiano & De Massis, 2015; Niehm et al., 2008; Zientara, 2017). The family and the firm are overlapping subsystems that mutually affect each other, so the question remains which family outcomes (e.g., family harmony, family well-being) may be achieved. Following Jaskiewicz and Dyer (2017), we ask to what extent these family outcomes act in subsequent phases as family antecedents.

- Research Question 2: Which family outcomes (i.e., family resources) can an owning family generate through the CSR activities of its firm, and how do those affect the family firm’s CSR activities in subsequent periods?

Although family outcomes were not explicitly examined, the research literature implicitly indicates that CSR-related family outcomes are generated through the use of firm outcomes (e.g., Aragón-Amonarriz et al., 2019; Campopiano & De Massis, 2015; Déniz and Suárez, 2005; Niehm et al., 2008; Zientara, 2017). It is a fundamental assumption of our SFBT-based theoretical framework that resources can be exchanged between family and firm as soon as the overlap of both subsystems is significant enough (Danes et al., 2008; Stafford et al., 1999), meaning the family firm has a unique resource base since it can draw from the owning family’s resources. Since the resource transaction between the two subsystems can also be performed from firm to family, this implies that the owning family can also benefit from the firm’s financial and non-financial outcomes of CSR.

However, as in the case of the antecedents, it is also necessary to consider where outcomes are concerned, that a subsystem’s boundary permeability can hinder the resource transfer effectiveness. For example, some studies examine the extent to which majority shareholders withdraw resources from a company at the expense of minority shareholders (Welford, 2007). This so-called ‘tunneling’ disadvantages minority shareholders, who, due to their limited influence, cannot protect themselves against majority shareholders (Dal Maso et al., 2020; Sahasranamam et al., 2020). Therefore, the potential for the transfer of firm resources (primarily financial or social capital) could lead to the firm subsystem decreasing its permeability to hamper resource flow to the family subsystem.

Although we propose putting greater emphasis on firm outcomes, we also propose that outcomes-related CSR research should include how family outcomes are affected by CSR. For example, it could be examined whether an increase in the firm’s performance through CSR also leads to an increase in the family’s well-being. Another suggestion would be to examine whether a firm image, improved through CSR, leads to more social capital for the owning family. In this regard, we also propose examining the extent to which conflicts occur between family and firm and to what extent this process influences the generation of family outcomes.

- Research Question 3a: Which firm outcomes (i.e., firm resources) affect the association between CSR activities and family outcomes (i.e., family resources)?
- Research Question 3b: Which conflicts can arise during the resource transaction between family and firm subsystem, and how does this affect the family firm’s CSR outcomes?

According to our SFBT-based theoretical framework, family and firm resources are used to overcome internal and external disruptions. Research has found that family firms are more sensitive to external contextual factors (Uhlaner et al., 2004) and more adaptive to them due to their unique set of resources. Research concerning CSR antecedents shows that stakeholder pressure and community embeddedness increase the likelihood that family firms will engage in CSR (Ge & Micelotta, 2019). The greater the pressure from contextual factors to engage in CSR, the more likely family firms are to mobilize their family resources for the firm (e.g., Berrone et al., 2010; Maggioni & Santangelo, 2017; Zamir & Saeed, 2020).

Research shows that this pressure varies significantly from region to region (Ertuna et al., 2019; Labelle et al., 2018). While it tends to be high in the USA and Europe, it tends to be low in Asian countries (Welford, 2007). However, the economic relevance of the Asian continent has increased, and the economic relationships between Asian countries and the Western world have become more relevant. Muttakin and Khan (2014) found that many Asian firms now use CSR to signal to foreign investors that they have more governance structures than other regional competitors (Cordeiro et al., 2018). Therefore, Asian family firms can use their family resources to enhance CSR and use it as a strategic tool to signal trustworthiness to Western investors (Du et al., 2018).

Contextual factors could affect the relationship between CSR activities and their outcomes. For example, different countries and communities may have different expectations of the owning family regarding CSR. Family firms could respond more effectively towards those expectations when expanding since they have a more significant resource base because of family resources. Therefore, we encourage future research to look for and examine contextual factors affecting the outcomes of CSR in family firms.

- Research Question 4: Which contextual factors affect the relationship between CSR activities and outcomes (i.e., family and firm outcomes) of family firms?

A crucial connection not yet addressed by current research involves CSR antecedents and defining which antecedents lead to which outcomes, also which CSR activities link those antecedents and outcomes. Whether the firm antecedents lead to firm outcomes or there are crossover connections due to the overlap of family and firm, so that, for example, firm antecedents generate family outcomes remains to be established. The effectiveness between family and firm antecedents is also an angle that should be considered.

Therefore, it is necessary to consider which, how, and why CSR activities link antecedents and outcomes. Labelle et al. (2018) theorize that economic and non-economic goals drive a family firm’s CSR activities. They argue that the higher the proportion of family ownership, the more likely the firm’s business activities align with achieving economic goals. Since they attribute a non-economic effect to CSR, they argue and deduce that more CSR is conducted in firms with lesser family ownership, and fewer CSR activities are conducted in firms with increased family ownership. Interestingly, Terlaak et al. (2018) theorize and empirically find the opposite by arguing that family firms place a higher emphasis on non-economic goals when family ownership within the firm increases.

Thus, since many family firms have scarce resources and must use them efficiently to survive (Ward, 1997), it is vital to understand which CSR activities will help them achieve the best results. Following Stafford et al. (1999) SFBT, a division of family and firm could help clarify these issues. Case studies could be used to identify relationships or disagreements between antecedents and outcomes. Their results could be checked quantitatively afterward using panel surveys to analyze the long-term effect of the measures. In future research, this black box must be opened to prove which CSR activities help achieve which goals and whether family resources can help achieve those.

- Research Question 5a: Which CSR activities (e.g., environmental, economic, or societal-related) are linked to which antecedents (i.e., family and firm antecedents) and outcomes (i.e., family and firm outcomes)?
- Research Question 5b: How and why do CSR activities link antecedents and outcomes of family firms?

A central goal of family firms is to ensure that the firm can continue to provide a basis for the family’s existence and even for later generations. While a handful of family firms achieve this goal, others do not (Koiranen, 2002). Among the outcome-related studies, Antheaume et al. (2013) found that CSR is a factor that positively influences the longevity of family firms, indicating that CSR helps family firms succeed over generations. In line with our SFBT-based theoretical framework, we found that substantial family influence leads to a greater propensity of CSR in family firms, which we trace back to family resources integrated within the family firm’s resource base. Those help the family firm to respond more effectively to internal and external disruptions and thus also to generate better outcomes out of CSR.

In this context, Pan et al. (2018) is particularly noteworthy since they find that CSR positively affects the family firm’s post-succession performance. They theorize that to take over successfully, successors of the owning family need to win the support of internal and external stakeholders, which they can do by conducting CSR (Bammens & Hünermund, 2020; Pan et al., 2018). Signaling good intentions to their stakeholders through CSR will increase the motivation of the firm’s stakeholders to interact (Bingham et al., 2011), helping to facilitate the social network transfer from the predecessor to the successor (Aragón-Amonarriz et al., 2019; Pan et al., 2018; Schell et al., 2020). Thus, CSR is a strategic instrument that increases the firm’s legitimacy (Chiu & Sharfman, 2011), consequently increasing the probability of a successful generational handover (Pan et al., 2018).

Accordingly, CSR could help a family firm preserve its resource base during the handover of the firm, thus contributing to the longevity of the firm as this is one of the most crucial issues of family firm research. Research empirically proving this assumption would create a business case for CSR in family firms. Therefore, this assumption must be addressed in future research.

- Research Question 6: How and why do CSR activities increase the longevity of family firms?

## Synthesis

### Discussion

This systematic literature review has revealed that CSR is still a relatively young phenomenon in family firm research but is becoming increasingly relevant. This review was guided by three research questions focusing on a family firm’s CSR antecedents and outcomes and their interaction. Using Stafford et al.’s (1999) SFBT to build our theoretical framework, we examined the CSR antecedents and outcomes of a family firm not only from a firm but also from a family’s perspective. We contribute to the literature by summarizing and integrating our findings in an over-arching framework, emphasizing family and firm antecedents, outcomes, and contextual factors. Thus, we uncover the current research focus on family firms’ CSR antecedents and outcomes (see Fig. 3) and show which research questions need to be addressed in the future (see Table 10). Our framework helps researchers to organize the existing research (e.g., Mariani et al., 2021) for a better understanding of this phenomenon and to address future problems and questions. In this regard, our review contributes to the further development of the research field.

Our review of the research literature shows that although CSR is a firm-level construct, CSR decision-making is not exclusively tied to the firm but also to the family subsystem and the family resources it provides (Dimov, 2017; Jang & Danes, 2013). The research literature provides evidence that increased CSR is implemented when the owning family strongly influences the family firm. Consequently, the use of family resources (i.e., familiness) to conduct CSR activities is more pronounced in smaller firms as it is more likely that an owning family will exert its influence in smaller firms than in larger ones (Danes & Brewton, 2012). From an SFBT point of view, this is the case since the owning family directs more family firm resources towards CSR activities from which it benefits doubly—firstly by the firm outcomes and secondly by the family outcomes. Thus, while not necessarily being more ethical than other firm owners, owning families are inclined to use resources provided by the family subsystem to conduct CSR on the firm level.

Also, according to SFBT, as family influence increases, family firms engage in CSR to cultivate their relationships with their stakeholders (Fitzgerald et al., 2010; Stafford et al., 1999), and thereby generate positive firm outcomes and longevity for the family firm by leveraging resources (Kuttner & Feldbauer-Durstmüller, 2018). As in the case of the antecedents-related studies, we consequently also examined the outcomes-related studies from a family and firm perspective. Concerning studies examining the firm outcomes of CSR in family firms, we found that the research focuses strongly on non-financial outcomes, whereas financial outcomes have rarely been researched. Regarding firm outcomes, we recommend that future research assign a higher priority to CSR’s financial firm outcomes. Family outcomes relating to the needs and goals of the owning family (Gómez-Mejía et al., 2007; Jaskiewicz & Dyer, 2017; Jaskiewicz et al., 2017) have not yet been analyzed at all, which is surprising, as the subsystems family and firm form a unit, and accordingly the outcomes should have a reciprocal influence (Stafford et al., 1999). Further research could pinpoint which family-related goals (Chrisman et al., 2010; Kotlar & De Massis, 2013) family firms can achieve through CSR.

While our literature review has shown that family influence increases the likelihood of CSR activities within family firms, consequently increasing the probability of achieving improved firm outcomes, we could not answer how CSR links both categories. Thus, the question concerning the catalytic role of CSR remains a black box. Since family firms need to know which antecedents can help them achieve their goals (i.e., family and firm outcomes) through CSR activities, this question needs to be answered. Family firms must invest the optimum set of family and firm resources into CSR activities, knowing that those investments will give them the strategic advantage they need; this is especially important for family-owned SMEs, which have considerably fewer resources available than their larger competitors.

In general, Jaskiewicz et al. (2017) called for a more robust integration of family science into this research area to better integrate the family as an organizational actor into management research. Family science uses knowledge coming “from various disciplines such as psychology, sociology, and education” (Jaskiewicz et al., 2017, p. 309) and, therefore, could provide new theoretical and empirical insights for the explanation of CSR’s family outcomes. Since it can be assumed that family firms do not conduct CSR purely out of altruism but also to achieve specific outcomes (Zientara, 2017), this area of research offers many opportunities for future family firm-related studies. Furthermore, a holistic theoretical framework such as Stafford et al.’s (1999) SFBT that considers the unity of family and firm as well as a permanent exchange of resources could be beneficial. This theory assumes that the resources are transferred between the family and the firm subsystem depending on the extent of the subsystems’ overlap (Danes et al., 2008; Fitzgerald et al., 2010). By identifying the underlying reasons for the interaction between family and firm, it might be possible to better explain a family firm’s CSR behavior.

### Practical Implications

The literature shows that family firms do indeed engage in more CSR. However, as the Waltons, Fords, Murdochs, and Sacklers of this world show, they do not necessarily do so because they are more ethical than non-family firms but rather because they achieve positive results for the family and the firm by doing so. Therefore, we must bear in mind that family firms are run according to business principles and consequently conduct CSR for the benefit of the family and the firm, and not necessarily for the benefit of society. If the lobby against unethical practices; child labor; slave labor; pollution; animal cruelty, for example, did not exist, would the chemical company continue to pollute the rivers, or the sweatshop stop using child labor? These are rhetorical questions but show CSR’s potential for improving the state of the world, on the one hand, and its limitations simultaneously.

Although we know that family firms use CSR activities, not for altruistic reasons but to benefit personally through family and firm outcomes, we should not forget the positive aspects of those activities for society. Given the severe social and environmental problems the world faces, it is crucial to motivate owning families to spend more resources on CSR activities helping to avoid or overcome those problems and compensate for any damages incurred while conducting their business. Thus, lobbies and regulating authorities, be they local or governmental, should consider how to encourage companies—family-owned or not—to behave with CSR and pursue ways and means to not only enhance their business, reputations, and profits but to behave in an ethical, sustainable manner at the same time.

Since the analysis of the research literature shows that family firms are more sensitive to contextual factors than non-family firms, more regulations for CSR activities can generate positive effects for society as a whole and for the family firm itself. In particular, CSR activities directly related to business (i.e., economic-related CSR) can benefit the firm. Whether a family firm or non-family firm and regardless of the positive effect of family influence and the motivation behind conducting the CSR activities, our study shows and research literature agrees that conducting CSR is a wise and far-sighted move for a company and a functional strategic tool a company can use to engender long-term profitability.

### Limitations

Following Tranfield et al.’s (2003) systematic literature review approach has helped us to expand the field of research, even if also accompanied by certain limitations. When using a selection of databases, there is the possibility that not all relevant papers have been considered. However, this limitation is counterbalanced partly by the detailed database description, making the analysis more comprehensible. Despite our systematic approach to searching and analyzing relevant publications, subjectivity cannot be entirely excluded. Nonetheless, this subjectivity has also helped us to identify lacuna and proffer essential questions, which we hope will open up future research on CSR in family firms. Also, we limited our literature search specifically to family firms. It is possible that there is research in the field of family science that further explores the effects between family and firm, as well as CSR activities, and that this review has not considered. Additionally, our chosen theoretical framework may impact the analysis and evaluation of the articles consulted. Accordingly, we clarified our basis of interpretation by explaining the theory and the underlying mechanism in detail.

Quantitative empirical approaches dominate research activities on CSR in family firms. To develop family firm-specific explanatory approaches for the influence of the family on firm antecedents and the function of translation from drivers to outcomes and the emerging dynamics, we encourage subsequent research to draw more on qualitative empirical research in the form of case studies and experiments for example (De Massis & Kotlar, 2014; Lude & Prügl, 2021). In particular, as research in family-owned SMEs is still under-represented (Miller & Le Breton-Miller, 2007), this approach should be conducted within family-owned SMEs. Research in the field of large companies cannot be transferred one-to-one to SMEs (Faller and zu Knyphausen-Aufseß, 2018; Uhlaner et al., 2012), as the involvement and integration of the family are different (Miller & Le Breton-Miller, 2007), leading to a different use of resources as well as goals (Block & Wagner, 2014b; Niehm et al., 2008). Qualitative empirical research could help fathom the underlying motivations of family firms concerning CSR outcomes. We also propose that such research focus more on the role of the owning family and its members. Research considering this could break down the current barriers of the research field and develop it further.

## Conclusion

We postulate that research on CSR outcomes is necessary to evaluate the effectiveness of family and firm antecedents. It can also provide further insight into the unity of the family and the firm, especially its use of resources to achieve specific goals. These results lead to a better understanding of the heterogeneity of family firms. Likewise, in future research, these approaches can be applied to non-family firms since, here, managers have a connection to the firms and can help determine the firm’s success through their use of resources such as social and human capital, which in turn enhances their reputation. With this literature review, we want to motivate researchers to continue looking at CSR from different perspectives.

## Appendix 1

See Tables 11, 12, 13.

**Table 11:** Content Analysis of Family Firm Specific Corporate Social Responsibility Antecedents

Authors (Year)	Antecedents	Outcomes	Theory	Method	Sample Size	Sample Firm Size	Country of Research	Key Findings
Abeysekera and Fernando (2020)	Family ownership; family management; founder name	–	Agency theory	Quantitative	232 firms (9 year panel)	L	USA	Family ownership and management are negatively related to CSR strength. If the family firm is named after the founder, this positively moderates the effect between family ownership and CSR concerns. During the financial crisis, family firms were associated with a lower CSR strength than non-family firms
Adams et al. (1996)	Family-firm status	–	–	Qualitative	444 firms (cross-sectional)	–	USA	Few differences exist in ethics-related behavior between family firms and non-family firms. Non-family firms, unlike family firms, have formal codes of ethics. Family firms pass on their ethical views informally through the corporate culture. Differences in ethical behavior arise from the company type
Agostion and Ruberto (2021)	Family ownership; stakeholder pressure	–	SEW, Stewardship theory, Institutional theory	Quantitative	–	–	International (Intercontinental)	This study provides evidence on the positive relationship between family firms and environment-friendly practices, also highlighting the positive moderating role of regulatory pressure
Amann et al. (2012)	Family ownership and management (combined)	–	–	Quantitative	200 firms (cross-sectional)	–	Japan	CSR ratings in terms of human resource management and environmental protection are higher for non-family firms than for family firms and there is no significant relationship between family firms/non-family firms regarding of CSR ratings of governance and social contribution
Amidjaya & Widagdo, 2020	Family ownership (also moderator); foreign ownership (also moderator) and corporate governance	–	Agency theory, Institutional theory	Quantitative	31 firms (5 year panel)	–	Indonesia	Family ownership, foreign ownership and corporate governance have a positive effect on sustainability reporting. Family ownership weakens the effect of corporate governance, foreign ownership has no moderating effect on corporate governance
Aragón-Amonarriz et al. (2019)	Family social capital; family commitment; family values	–	–	Qualitative	3 firms (13 interviews)	S M	Mexico	In order to maintain responsible family ownership across generations, family firms must preserve their family social capital, consisting of a cognitive, structural, and relational dimension
Arena and Michelon (2018)	Family values; family control; family identity; firm age	–	SEW	Quantitative	167 firms	–	Italy	High levels of the family control and influence lead to lower environmental disclosure compared to non-family firms, with the effect weakening over the life cycle. Middle-aged family firms with high family identity provide more environmental disclosure, for example to protect and enhance the reputation and image of the business, whereas old family firms with high family identity provide less environmental disclosure
Bammens and Hünermund (2020)	Family ownership (mediated by company reputation motive); family values and transgenerational intention (moderator family ownership-reputation)	–	Institutional theory	Quantitative	4009 firms (cross-sectional)	S M L	Germany	Family ownership has a positive effect on the introduction of eco-innovations, partly due to the focus on the company reputation (mediator). This effect is highest when the family has transgenerational intentions
Baù et al. (2021)	Internationalization; local roots	–	–	Conceptual	–	–	–	This editorial of the special issue on ‘Locality and Internationalization of Family Firms’ discusses how family firms can build bridges between communities increasingly drifting apart. By bridging local and global environments, family firms can contribute to the sustainable development of the society
Bendell (2022	family ownership and management; governmental pressure; industry pressure	–	Stakeholder theory	Quantitative	121 firms	–	USA	The study’s results demonstrate that family firms who were highly engaged with their peers were significantly less influenced by the possibility of negative peer reputation repercussions when making environmental innovation investment decisions compared with other firms
Bennedsen et al. (2019)	Family management	–	–	Quantitative	2600 firms (5 year panel)	S M L	Denmark	Company characteristics, such as its policy/environment and incentives and corporate culture have a strong influence on employee absenteeism. Likewise, employee selection has a small influence on absenteeism. Overall, family firms have lower employee absenteeism
Bergamaschi and Randerson (2016)	Family ownership	–	–	Theoretical	–	–	–	A family firm can be divided into the three subsystems family; ownership; and business. Depending on which subsystem is the dominant one, the family firm will follow different CSR patterns
Berrone et al. (2010)	Family ownership; family control	–	–	Quantitative	194 firms (4 year panel)	–	USA	Family firms have a better environmental performance to protect their SEW compared to non-family firms. This is particularly the case at the local level. This effect is independent of whether the family member serves as CEO, or CEO and board chair
Bhatnagar et al. (2020)	Family values; family religion	–	SEW, Agency theory	Qualitative	14 firms (24 interviews)	–	India	The Hindu beliefs of Dharma (duty to society) and Karma (right to act without expecting rewards) influence CSR-related philanthropy of family firms
Bingham et al. (2011)	Family involvement; founder involvement	–	Stakeholder theory, Organizational identity theory	Quantitative	706 firms (15 year panel)	L	USA	Family firms demonstrate more CSP social initiatives than non-family firms, This effect becomes even greater with increasing family and/or founder involvement
Biswas et al. (2019)	Family ownership; corporate governance (mediator)	–	–	Quantitative	(16 year panel)	–	Bangladesh	The relationship between corporate governance guidelines and CSR reporting is mediated by the quality of corporate governance. Since family firms have a lower quality of corporate governance, they also have lower levels of CSR reporting
Block (2010)	Family ownership; family management	–	Social identity theory, Agency theory	Quantitative	414 firms (9 year panel)	L	USA	Family firms downsize less than non-family firms. The decisive factor is whether the company is family-owned or family-managed, as the positive effect can only be demonstrated in family-owned companies. Compared to non-family firms, family firms only downsize when this is necessary to protect their employees, and thus act more socially responsibly than non-family firms
Block and Wagner (2014a)	Family ownership; family management	–	SEW	Quantitative	399 firms (9 year panel)	L	USA	Family ownership as well as founder ownership reduces CSR concerns, whereby founder ownership has a stronger influence. Family management, as well as founder management, increases CSR concerns. In this case, family management has the stronger effect
Block and Wagner (2014b)	Family ownership	–	–	Quantitative	286 firms (11 year panel)	L	USA	Family ownership has a positive effect on diversity-, employee-, environmental-, and product-related aspects of CSR performance and a negative impact on community-related CSR. The latter is a consequence of the owner family supporting the community through private rather than business channels
Blodgett et al. (2011)	Family values; ethical values	–	–	Quantitative	172 firms (cross-sectional)	–	International (Intercontinental)	Comparing mission statements, U.S. family firms have more ethical values than international family firms and non-family firms in the U.S.. U.S. family firms focus on “integrity” and “honesty”, while international family firms focus on “environmentalism”, “globalism”, and “CSR”. An increase in the ethical values of family firms around the world has occurred over time
Cabeza-García et al. (2017)	Family ownership; governance; foreign ownership (moderator)	–	SEW	Quantitative	105 firms (7 year panel)	L	Spain	Family firms have a lower commitment to CSR reporting than non-family firms. With regard to the second-largest shareholder, foreign ownership moderates this effect negatively, while the presence of a second family even increases this effect
Campopiano et al. (2014)	Family ownership; family management	–	Stewardship theory	Quantitative	130 firms (cross-sectional)	S M	Italy	Family ownership has a positive effect on the company’s philanthropic involvement, while family involvement in management has a negative effect. Family owners invest more in their business to build reputation and be a better steward in the community to support the longevity of the business
Campopiano and De Massis (2015)	Family ownership and family management; institutional setting; community embeddedness		Institutional theory, Grounded theory	Qualitative	98 firms (cross-sectional)	M L	Italy	Through CSR, family firms try to positively enhance their reputation. For this reason, they are less influenced by institutional settings and focus more on the expected social outcomes of CSR. Since they focus CSR less on institutional requirements and more on the interests of the stakeholders to be influenced, the variance of their CSR reports is higher
Campopiano et al. (2019)	Family management	–	Self-construal theory	Quantitative	63 family firms (cross-sectional)	L	International (Intercontinental)	Female members on the board of family businesses exert a positive influence on CSR if they are not family members of the controlling family. In contrast, they only have a positive influence on philanthropic engagement if they are family members of the controlling family
Chen and Cheng (2020)	Family ownership and/or management (combined); mimetic pressure from industry (moderator)	–	Agency theory, Neo-institutional theory	Quantitative	(4-year panel)	L	Taiwan	Public family firms acquire CSR assurances less frequently than non-family firms. This relationship is positively moderated by mimetic pressure from industry peers
Chen and Liu (2022)	family ownership and management; culture type (moderator)	–	SEW	Quantitative	58 papers (meta-analysis)	–	International (Intercontinental)	The study’s findings show evidence of greater CSP among family firms compared to non-family firms. The family firm–CSP relationship was moderated by cultural values such as ingroup collectivism, humane orientation and future orientation, and the moderating effects depended on cultural tightness
Cordeiro et al. (2018)	Family ownership	–	Neo-Institutional Theory	Quantitative	500 firms (4 year panel)	L	India	Family ownership and multinational ownership have a positive impact on a firm’s CSR rating. State ownership, on the other hand, leads to a decline in CSR ratings
Cordeiro et al. (2020)	Board gender diversity; family ownership (moderator)	–	SEW, Agency theory, Resource dependency theory	Quantitative	751 firms (6 year panel)	L	USA	Board gender diversity is positively associated with corporate environmental performance. This relationship is positively moderated by family ownership, but also by being a dual-class firm
Cruz et al. (2014)	Family ownership and management (combined); national CSR standards (moderation); industry CSR standards (moderation); declining performance (moderation)	–	Organizational identity theory, SEW, Stakeholder theory	Quantitative	598 firms (4 year panel)	L	International (Europe)	Family firms conduct more CSR towards external stakeholders, less CSR towards internal stakeholders, and are at the same time less sensitive to national CSR standards or those of their industry (moderation). Family firms place their corporate survival above their SEW and their CSR activities are therefore more sensitive to declining performance than those of non-family firms (moderation)
Cruz et al. (2019)	Family management; non -family/family female directors	–	–	Quantitative	152 firms (5 year panel)	L	USA	Women in boards of family firms affect CSP positively. This effect can be observed for outside non-family and inside family women directors
Cuadrado-Ballesteros et al. (2015)	Independent directors; family ownership (moderator)	–	–	Quantitative	575 firms (7 year panel)	–	International (Intercontinental)	Due to a higher proportion of independent directors in a firm’s board, a firm discloses more CSR. Owning families, on the other hand, use their position to influence independent directors to make fewer CSR disclosures. Thus, family ownership negatively moderates this relationship
Cuadrado-Ballesteros et al. (2017)	Family ownership; formal code of ethics	–	–	Quantitative	547 firms (9 year panel)	–	International (Intercontinental)	Due to less formalization, family firms tend to use less formal codes of ethics than non-family firms, which mediates the negative relationship between family ownership and CSP
Cui et al. (2018)	Family ownership; family management (moderator); long-term incentive compensation (moderator)	–	Behavioral agency theory	Quantitative	177 firms (8 year panel)	L	USA	Family management positively moderates the positive effect of family ownership on the CSR performance. Family and non-family CEOs are incentivized to increase CSR through long-term incentive compensation (moderation), although the effect is lower for family CEOs than for non-family CEOs
Dal Maso et al. (2020)	Family ownership; human resource practices (mediator)	–	Agency theory	Quantitative	4932 firms (14 year panel)	–	International (Intercontinental)	Listed family firms have lower environmental performance than non-family firms. The study shows that this negative effect is mediated by a lower investment in employee training and development practices. This is due to a stronger bargaining power of the dominant coalition and a lower firm performance. Due to less investment in training and development
Dawson et al. (2020)	Family management; family generation	–	Signaling theory, Ability perspective, Willingness perspective	Quantitative	161 family firms (2 year panel)	S M	Italy	Business legality increases with the level of family involvement in management. Likewise, the level of generation has a positive influence
Dayan et al. (2019)	Family values; firms capabilities (mediator)	–	Mindfulness theory	Quantitative	150 firms (cross-sectional)	–	United Arab Emirates	Mindfulness in protection the SEW dimensions “identification of family members with the firm” and “binding social ties” influences the environmental strategy of family firms. They have a positive effect on production of sustainable products and processes. This effect is positively mediated by the capabilities of the company
Dekker and Hasso (2016)	Family-firm status; social embeddedness (moderator)	–	–	Quantitative	1452 firms (longitudinal)	S M	Australia	Private family firms have a lower environmental orientation than non-family firms. By contrast, when family firms are strongly embedded in the social community, they exhibit a higher level of environmental orientation
Delmas and Gergaud (2014)	Family values (heir succession intention; moderator of quality motivation, market motivation, and eco-certification)	–	Stakeholder theory	Quantitative	281 firms (cross-sectional)	–	USA	Using wineries as examples, it is shown that the intention to pass on the family business to the next generation has a positive effect on the adoption of eco-certificates. In this context, passing on the business moderates the effect of market motivation and quality motivation on eco-certification positives
Déniz and Suárez (2005)	Family value	–	–	Quantitative	112 firms (2 year panel)	S M	Spain	Depending on the owning family’s values, family firms tend to adopt a classic, socio-economic, or philanthropic approach to CSR. Most family firms in the sample followed a philanthropic approach, through which they tried to maintain broad relationships with society
Dick et al. (2021)	family ownership and management; overconfidence (moderator)	–	SEW	Quantitative	343 (cross-sectional)	M	Poland	This study demonstrates that founder-controlled family firms show low levels of CSR engagement. Moreover, overconfident executives in these firms tend to exhibit superior CSR performance
Dou et al. (2019)	Family ownership; family commitment (moderator); long term orientation (mediator)	–	Strategic reference point theory, Organizational identity theory, SEW	Quantitative	454 family firms (cross-section)	–	China	In family-owned businesses, commitment and long-term orientation is needed to implement a proactive environmental strategy. In family-owned businesses, commitment and long-term orientation is needed to implement a proactive environmental strategy. The mediation effect of long-term orientation is only significant if the level of family commitment is high
Discua Cruz (2020)	Family values; family religion	–	Stewardship theory, Paradox theory	Qualitative	1 firm (interviews)	M	Honduras	In line with their family values and religion, the owning family of Honduran firm AsphaCo frequently engaged in CSR activities towards the community and employees. Since they did not engage in CSR to better represent their firm, they preferred to keep it anonym and not report it publicly
Du (2015)	Corporate environmental misconduct; CEO’s political network (moderator)	–	–	Quantitative	3008 family firms (cross-sectional)	–	China	Family-owned firms use corporate philanthropic giving to distract from their corporate environmental misconduct. Since politically well-connected CEOs can avoid high personal penalties due to their political contacts, this effect is negatively moderated by the CEO’s political network
Du et al. (2016)	Media coverage; family ownership (moderator)	–	Institutional theory, Stakeholder theory	Quantitative	733 family firms (7 year panel)	–	China	Due to the higher visibility that comes with more media coverage, managers of family firms conduct more philanthropic giving. As the dependency on stakeholders decreases with family ownership, there is a negatively moderating effect of family ownership between media coverage and corporate philanthropic giving
Dyer and Whetten (2006)	Family ownership and management (combined)	–	Self-interest theory, Social identity theory	Quantitative	261 firms (10 year panel)	L	USA	In terms of social initiatives there are no differences between family firms and non-family firms. Family firms are more likely to avoid social concerns than non-family firms
El Ghoul et al. (2016)	Family ownership	–	Agency theory	Quantitative	335 firms (10 year panel)	–	International (Asia)	In order to achieve personal benefits, increasing family ownership allows family owners to divert the firm resources to activities that bring them financial benefits. Furthermore, this is particularly true for family firms with greater agency problems and from countries with weak institutions
El-Kassar et al. (2018)	Audit committee; family management (moderator)	–	Stakeholder theory, Agency theory	Quantitative	203 employees of family firms (cross-sectional)	Employees	Lebanon	Audit committees have a positive impact on CSR practices in the areas of health, refugees, community, and environment. In addition, family management has a positive moderating effect on the impact of CSR towards community, the environment, and health
Ertuna et al. (2019)	Family values; state regulations	–	Institutional logics theory	Qualitative	2 firms (case study)	–	Turkey	This study compares the CSR logics of a local hotel and a hotel chain. The local hotel adapts its CSR logic to local conditions and embeds it in the organization. The CSR logic of the hotel chain, which is specified by the headquarters, is only partially adopted and varies in its interpretation and implementation. Local needs and priorities are only implemented to a limited extent. The sustainability strategies in both companies are shaped by the family or the family headquarters
Fitzgerald et al. (2010)	Attitude towards community; community vulnerability; family firm resources	–	Sustainable family business theory	Quantitative	334 family-firm households (2 year panel)	S M	USA	Members of the owning family with a very positive attitude towards their local communities were more willing to support their local community. Those with higher education were more likely to do so by taking leadership positions in the community, while individuals with more household assets and profit companies were more likely to provide financial assistance to their communities
Fritz et al. (2021)	Family ownership and management	–	Institutional theory	Qualitative	12 firms	S M L	France	They find that family firms take a specific approach in supply chain management, especially that the social sustainability dimension is crucial for family firms. For non-family firms, the focus is on the ratio of costs to benefits and client satisfaction
García-Sánchez et al., (2021	Hostile environmental conditions (moderatred by family ownership and management)	–	SEW, Stakeholder theory	Quantitative	956 firms (9 year panel)	L	International (Intercontinental)	This study shows that family-controlled firms adopt CSR strategies and balance the demands of internal and external interest groups to preserve their SEW while facing fierce competition, resource scarcity, and penurious economic conditions
Gallo (2004)	Family-firm status	–	–	Quantitative	44 academics (cross-sectional)	People working on university institutions	International (Intercontinental)	Family firms fulfill their responsibility to create wealth and provide products for society to a high degree. But there must be a greater focus on sustainable and long-term execution, otherwise it will only be generated for one generation
Ge and Micelotta (2019)	Firm visibility; political linkages; family ownership and management (moderator)	–	–	Quantitative	3075 firms (cross-sectional)	–	China	Firms that are more sensitive to institutional pressure due to firm visibility and political linkages are more likely to engage in philanthropy and further donate larger amounts. While family ownership has no effect on the giving behavior, it does moderate the relationship between institutional pressure and the amount of philanthropic giving positively
Graafland et al. (2003)	Family-firm status; firm size	–	–	Quantitative	111 firms (cross-sectional)	S L	Netherlands	Family firms and non-family firms show very similar patterns regarding to the use of instruments such as codes of conduct, ISO certification, social reporting, social handbooks, and confidential. These instruments are influenced by the size of the company
Graafland (2020)	Family ownership; family management; firm size (moderator)	–	SEW	Quantitative	3816 family firms (cross-sectional)	S M L	International (Europe)	The relationship between ownership of a family firm and environmentally friendly production is stronger for smaller than for larger firms and is moderated by the involvement of family members in the management of the firm in a non-linear way. Differences between family and non-family firms are greater in small firms with family and non-family managers. Likewise, the best environmental performance is achieved in family firms managed by families and non-family members
Huang et al. (2009)	Family ownership and management (moderator: negative (regulatory stakeholder pressure; market shareholder pressure); positive (pressure from internal stakeholders)	green technical innovation/green administrative innovation	–	Quantitative	235 firms (cross-sectional)	–	Taiwan	Using the chemical, electronics and information technology industries, it is shown that there is a positive correlation between the degree of natural environmental pressure from stakeholders and the introduction of green innovations. The moderating effect of family firms leads to a negative effect of regulatory stakeholder pressure and market shareholder pressure on green innovations and a positive one of pressure from internal stakeholders. These differences are explained by organizational culture and core values
Iyer and Lulseged (2013)	Family ownership and management (combined)	–	Agency theory, Legitimacy theory, Stakeholder theory	Quantitative	397 firms (cross-sectional)	L	USA	There is no statistically significant difference in the probability of CSR disclosure (sustainability reporting) between family firms and non-family firms
Kallmuenzer et al. (2018)	Family values; community embeddedness/stakeholder pressure	–	Random utility theory	Quantitative	152 firms	S M	Austria	Family firms in rural tourism are motivated by environmental and social considerations after a financial security, which gives a greater benefit than a greater financial profit, through the family conditional SEW dynamics and the resulting CSR increase
Kariyapperuma and Collins (2021)	SEW	–	Family logics	Qualitative	72 firm websites	S M	New Zealand	Antecedents of heterogeneities (i.e., family goals, family values, culture and ethics, and the imprints of the founders and the next generation) were revealed with a discussion of three typologies of family firms: family first, business first and upstarts
Kim and Lee (2018)	Family ownership and management (combined); family CEO; family firm type	–	Agency theory	Quantitative	200 firms (3 year panel)	S M L	South Korea	Family firms have a lower CSP than non-family firms, which is even lower when a family firm is managed by a family CEO. Chaebols (family-run or controlled conglomerate) have a higher CSP than non-chaebol firms
Kim et al. (2017)	Top management attention to the natural environment; family ownership and management (combined) (moderator)	–	Behavioral theory, Institutional theory	Quantitative	97 firms (10 year panel)	–	USA	Family influence has a positive moderating effect on the relationship between top management’s attention to the natural environment and proactive environmental action
Kim et al. (2020)	Family ownership; population size (moderator)	–	Place theory	Quantitative	2000 firms (14 year panel)	L	USA	Family ownership reduces the probability of layoffs. The size of the population of the location in which the company operates has a negative moderating effect on this relationship
Labelle et al. (2018)	Family ownership; family control; stakeholder/shareholder-oriented economy	–	SEW, Agency theory	Quantitative	1264 firms (cross-sectional)	–	International (Intercontinental)	Family firms have a lower CSP than non-family firms. In family firms, family control (voting rights) up to 36% increases the CSP in family firms, whereas family control more than 37% decreases their CSP. Family firms operating in stakeholder-oriented countries pay more attention to social issues than those operating in more shareholder-oriented countries
Lamb and Butler (2018)	Family ownership; family management; founding family presence	–	Stewardship theory, SEW, Multiple agency theory	Quantitative	153 firms (13 year panel)	Fortune 500	USA	Family ownership and the presence of a family CEO increase CSR strength. In terms of CSR concerns, the presence of a family CEO and a founding family has a negative effect
Le Breton-Miller and Miller (2016)	Being a family firm	–	–	Conceptual	–	–	–	Characteristics that positively influence sustainability in family firms are: long-term orientation, reputation and agency costs. A negative effect can result from the following factors: family conflicts, SEW, and exploitation of smaller shareholders at the expense of sustainability. Important mediating roles (both positive and negative) could be family values, educational background, organizational factors, governance arrangements and environmental forces
López-González andet al. (2019)	Family ownership; family management (moderator); market munificence (moderator)	–	SEW, Agency theory, Stakeholder theory, Institutional theory	Quantitative	956 firms, (9 year panel)	L	International (Intercontinental)	Family ownership has a positive effect on CSR performance. This relationship is positively moderated by family management and family directors on the board of directors, but negatively moderated by high market munificent
Ma et al., (2022	Family ownership; family management	–	SEW	Quantitative	2671 firms (9 year panel)	L	China	This study reveals that SEW will enhance environmental responsibility when a family member serves as the company’s chairman. There is a significant inverted U-shaped relationship between the length of family involvement and corporate environmental responsibility
Madden et al. (2020)	Family ownership; Family firm age (moderator)	–	Socioemotional selectivity theory	Quantitative	1436 firms (8 year panel)	L	USA	Family ownership positively affects investments in CSR activities. As family firms age, they become more selective and invest less heavily in CSR activities. Family firms age acts as a moderator of the relationship between family ownership and CSR
Maggioni and Santangelo (2017)	Environmental non-profit organizations (substitutes for environmental regulations)	–	Stakeholder theory, Organizational science	Quantitative	2275 firms (cross-sectional)	S M L	Italy	Environmental non-profit organizations operating in the same local context influence family businesses more than non-family businesses to implement more green investment strategies. This is due to their scarcity of resources, risk aversion and local roots, which makes family businesses more sensitive to their pressure. Environmental non-profit organizations can serve as a substitute for sustainability compliance in sectors with low regulation
Marques et al. (2014)	Family involvement; family values	–	Stewardship theory, SEW	Qualitative	12 firms (interviews)	–	Spain	In family firms with family CEOs, but more frequently in family firms with a high family ownership, the values of identification and commitment are particularly evident. These have a strong impact on workplace- and community-related CSR
Martínez-Ferrero et al. (2018)	Family ownership; managerial discretion	–	Agency theory	Quantitative	1275 firms (8 year panel)	–	International (Intercontinental)	Family firms are taking more CSR measures to meet stakeholder demands and thereby gain greater discretionary power, for example to compensate for the manipulation of profits
Martínez-Ferrero et al. (2017)	Board size; board independency; family ownership (moderator)	–	Agency theory, Institutional theory	Quantitative	536 firms (8 year panel)	–	International (Intercontinental)	In stakeholder-oriented countries, board size and board independence have a positive effect on sustainability assurance, which is further positively moderated by family ownership. Board size and board independence also have a positive effect on sustainability reports provided by professional accountants, which are not influenced by family ownership
McGuire et al. (2012)	Family ownership and family management; corporate governance (moderator)	–	–	Quantitative	473 firms	L	USA	Family firms are less likely than non-family businesses to engage in negative or socially harmful activities. In general, corporate governance is not related to the social performance of the company. However, it has a moderating effect on the relationship between family control and social performance
Memili et al. (2020)	Family ownership; long term orientation (moderator)	–	Psychological capital theory	Quantitative	192 family firms (cross-sectional)	S M	Turkey	Using data of the hospitality and tourism industry, it is shown that SEW has a negative effect on firm performance (sales) and that family firm psychological capital mitigates this effect. Likewise, this effect can be mitigated by a long-term orientation, if non-financial strengths (family firm psychological capital) are used and the effects of financially focused goals are minimized
Meier and Schier (2021)	Family management	–	Behavioral agency model	Quantitative	555 firms (4 year panel)	L	International (Intercontinental)	This study provides evidence that family CEOs are positively associated with both external and internal CSR, whereas non-family CEOs within family firms tend to be negatively associated with both external and internal CSR
Miroshnychenko et al. (2022)	Family ownership	–	Agency theory, Stewardship theory	Quantitative	26 articles (meta-analysis)	S M L	International (Intercontinental)	This study concludes that the average effect of family involvement on environmental performance is negative, albeit small
Muttakin and Khan (2014)	Family ownership	–	Legitimacy theory	Quantitative	135 firms (5 year panel)	L	Bangladesh	Family ownership has a negative effect on CSR disclosures. Export oriented sectors, firm size and industry sectors also have a positive effect on CSR disclosures
Nadeem et al. (2020)	Family ownership; board gender diversity	–	Stakeholder theory Gender socialization theory	Quantitative	399 firms (10 year panel)	L	UK	Board gender diversity has a positive effect on stakeholder value creation. This effect can be observed for economic, social and environmental performance. Even though female directors of family firms are associated with environmental value creation, they have no impact on economic and social value creation
Oh et al. (2019)	Family management; outside directors’ ownership (moderator); board educational diversity (moderator)	–	–	Quantitative	290 firms (5 year panel)	L	South Korea	Outside directors’ ownership, as well as board educational diversity, have a positive moderating effect on CSR in firms with low family management but a negative moderating effect on CSR in firms with high family management
Palma et al. (2022)	Family ownership; family management; billionair ownership	–	Legitimacy theory, SEW	Quantitative	360 (cross-sectional)	–	International (Europe)	This study reveals that greater prominence is attributed to sustainability issues on corporate websites when the family company’s CEO is a family member and the level of family ownership is lower. The prominence attributed to sustainability issues on corporate websites by family companies owned by European billionaires is greater than that of family companies not owned by billionaires, but only when using a less stringent measure of prominence
Peake et al. (2017)	Owning family’s duration in a community; owning family’s community satisfaction; female management (moderator)	–	Social capital theory	Quantitative	279 family-firm households (2 year panel)	S	USA	The owning family’s duration in a community is positively associated with their participation in community development activities. The greater the level of dissatisfaction with the community, the greater the willingness to participate in the work of supporting community development. Female management moderates the relationship between community satisfaction and participation in community development
Rees and Rodionova (2015)	Family ownership; liberal/coordinated market economy	–	–	Quantitative	3893 firms (11 year panel)	–	International (Intercontinental)	Closely held equity and family ownership have a negative impact on environmental, social, and governance performance. In terms of governance control, closely held equity shows no impact on environmental and social rankings, whereas family ownership continues to have a negative impact. These results are found in liberal market economies and coordinated market economies, whereas the latter generally reflects weaker performance and considerable diversity
Richards et al. (2017)	Family influence; domestic world or civic-green world rhetoric, multi-generation control (whether one family had been the firm's main blockholder for at least two generations)	–	–	Quantitative	86 firms (cross-sectional)	–	International (Intercontinental)	Using the coffee, tea, and chocolate industry as an example, the study shows that investment in sustainability certificates depends on the chosen legitimacy principles and the reflection of identity orientation, the “civic and green” world and the “domestic” world. The domestic dependency of the companies has a negative impact on investments for sustainability certifications, because the legitimacy communication takes place in a different way. The methods of legitimacy depend on the generational integration of the family firm. Multi-generational family firms can better integrate socially responsible attributes through its long-term stakeholder relationships and therefore do not need sustainability certifications
Sahasranamam et al. (2020)	Family ownership; business group owned (moderator)	–	Agency theory, Institutional theory	Quantitative	(8 year panel)	L	India	Family ownership is positively related to community-related CSR. There is no significant moderating effect of business group ownership in this relationship
Samara et al. (2018)	Family ownership; family management; family commitment; generation of management; outside directors	–	SEW	Qualitative	146 family firms (cross-sectional)	-	International (Intercontinental)	Family firms show positive SEW and higher environmental social performance when 100% of the business is in family hands, when the first generation is still involved in management, when the board is dominated by family members, and when family involvement in management is low. When many family members are involved in management, the negative effects of SEW become visible and less social performance is performed. Non-Anglo-Saxon countries focus more on long-term stakeholder welfare and less on managerial compensation to firm profitability. Therefore, they achieve high social performance (a) when family-dominated management is complemented by a board of family and non-family members, (b) when business ownership is shared with external parties and non-family board members mediate conflicts between them, and (c) when there is shared business ownership and the top management team is dominated by the first generation
Sánchez-Medina and Díaz-Pichardo (2017)	Family values; environmental values (mediator); environmental pressure	–	Sustainable family business theory	Quantitative	72 family firms (cross-sectional)	S	Mexico	Environmental pressure leads to the introduction of quality practices in artisanal family firms through the formation of environmental values. This effect is completely mediated by the environmental values of the owner family
Seckin-Halac et al. (2021)	family ownership; Ownership concentration	–	Agency theory, Stakeholder theory, Theory of planned behavior	Quantitative	4479 firms (6 year panel)	L	International (Intercontinental)	The study finds by a moderated path analysis that family shareholding weakens the direct effect of ownership concentration on board gender diversity and its indirect effect on corporate social responsibility
Sharma and Sharma (2011)	Family influence; family values; relationship conflict (negative moderator)	–	Theory of planned behavior	Conceptual	–	–	–	High family involvement has a positive effect on the exercise of the proactive environmental strategy. This intention is increased if there are low relationship conflicts within the controlling family in the family business, as resources for implementation can then be better provided
Terlaak et al. (2018)	Family ownership; family management	–	SEW	Quantitative	259 firms (7 year panel)	L	South Korea	Family ownership and the propensity of a business group to disclose environmental performance information have a U-shape relationship. This U-shape is moderated and strengthened by the presence of a family CEO
Uhlaner et al. (2004)	Family firm status; family generation; community embeddedness; firm size; family firm name includes family’s surname	–	Stakeholder theory	Quantitative	42 family firms (cross-sectional)	S M	Netherlands	The family character of the business most frequently impacts employee, client, and supplier relationships. Statistically significant interaction effects are reported for the following moderator variables: generation of the owner; company tenure in the community; community size; company size; and inclusion of the family surname in the business name
Uhlaner et al. (2012)	Family influence (power, experience, culture); number of owners (moderator)	–	Theory of planned behavior	Quantitative	689 firms (2 year panel)	S M	Netherlands	SMEs with a greater family influence are more likely to engage in environmental management practices. This increases in family firms with larger business-owning families (moderation)
Wiklund (2006)	Family ownership	–	Agency theory	Conceptual	–	–	–	Family firms show more positive CSR behavior, due to the bond between the family and the company
Yu et al. (2015)	Family ownership; majority ownership; independent directors	–	SEW	Quantitative	229 firms (5 year panel)	–	Taiwan	Family firms have a better CSR performance than non-family firms. SEW, measured by the majority shareholding and the proportion of independent directors on the board, has a positive effect on CSR
Yu et al. (2021)	Family ownership and management; provincial environmental regulations	–	Agency theory	Quantitative	(6-year panel)	L	South Korea	This study shows that family firms with ownership, operational, and strategic control can achieve higher environmental performance within a province with more stringent environmental regulations
Zamir and Saeed (2020)	Closeness to financial markets; family ownership (moderator); family commitment	–	Legitimacy theory Institutional theory	Quantitative	649 firms (6-year panel)	–	International (Intercontinental)	Firms located closer to the financial centers have a higher CSR disclosure rates compared to their more distant counterparts. This effect is positively moderated by family ownership and being a listed company. The negative effect of distance on CSR disclosure is stronger in countries with higher income inequality
Zheng et al. (2017)	Family values; Machiavellian corporate culture (low organizational justice, psychological contract violation, low trust)	–	Social information processing theory Grounded theory	Qualitative	Single case study	L	China	The study examines why employees exhibit counterproductive work behavior. This is caused, among other things, by Machiavellian corporate culture, which is characterized by low trust and strong control, as well as psychological contract violations, as these factors leads to the idea of mutual exploitation

*S* small, *M* medium, *L* large (listed)

**Table 12:** Content Analysis of Family Firm Specific Corporate Social Responsibility Outcomes

Authors (Year)	Antecedents	Outcomes	Theory	Method	Sample Size	Sample Firm Size	Country of Research	Key Findings
Adomako et al. (2019)	–	Firm performance (insignificant)	Resource-based view theory	Quantitative	253 firms (cross-sectional)	S M	Ghana	Environmental sustainability orientation shows no significant impact on performance in family businesses, but is enhanced in non-family businesses
Hsueh (2018)	–	External non-financial outcomes	Source credibility theory	Quantitative	Study 1: 167 NGO’s (cross-sectional), Study 2: 335 stakeholder	–	International (Intercontinental)	Family firms have a larger credibility gap in publishing sustainability reporting than non-family businesses. This gap can be reduced by an independent assurance service. Family businesses derive greater value with reasonable assurance than non-family firms
Lin et al. (2020)	–	Credit rating (moderated by family ownership)	Agency theory	Quantitative	1475 firms (9 year panel)	L	Taiwan	CSR has a moderating and partial mediation effect between corporate governance and credit rating. However, this connection could not be established for family firms
Martínez-Ferrero et al. (2018)	–	Information asymmetry (moderated by family ownership)	Agency theory	Quantitative	548 firms (7 year panel)	–	International (Intercontinental)	CSR disclosures reduce information asymmetry, which in turn also positively effects the level of CSR disclosures. Family ownership has a negative moderating effect on the relationship between CSR and information asymmetry, reversing the original negative effect. The effect between information asymmetry and CSR is negatively moderated by family ownership, reversing the original effect in family firms
Maung et al. (2020)	–	Financial market reaction (moderated by family management)	Signaling theory	Quantitative	(10 year panel)	–	USA	The financial markets react positively to the donations of religious CEOs and are further positively moderated by is the presence of a founder or family CEO
Naciye Sekerci et al. (2022)	–	Investors reaction (moderated by family ownership and management)	Signaling theory	Quantitative	133 firms (11 year panel)	L	France	Markets react more positively to CSR news from family firms than from non-family firms
Pan et al. (2018)	–	Financial outcomes; post-succession performance	–	Quantitative	885 firms (9 year panel)	–	China	Family firms exhibit more corporate philanthropy when the handover to the second generation is imminent. In the process, better market and accounting performance is achieved in addition to generally poor performance, indicating a strategic deployment
Panwar et al. (2014)	–	External non-financial outcomes	–	Quantitative	278 US residents (cross-sectional)	–	USA	Firm outsiders perceive the legitimacy of CSR measures of family-owned firms as higher than those of publicly listed firms
Samara and Arenas (2017)	–	Internal non-financial outcomes; long-term family firm survival and success; firm reputation	–	Conceptual	–	–	–	Family firms that promote fairness in the workplace can benefit by preserving their reputation and enhancing the long-term survival of the business
Wagner (2010)	–	Innovation with high social benefits (moderated by family ownership)	–	Quantitative	252 firms (11 year panel)	L	USA	There is a positive link between CSP and innovation with high social benefits. This relationship is positively moderated by family ownership

*S* small, *M* medium, *L* large (listed)

**Table 13:** Content Analysis of Family Firm Specific Corporate Social Responsibility Antecedents and Outcomes

Authors (Year)	Antecedents	Outcomes	Theory	Method	Sample Size	Sample Firm Size	Country of Research	Key Findings
Ahmad et al. (2020)	Family involvement (family commitment; family continuity; family control; family enrichment)	Financial outcomes (financial strength); internal non-financial outcomes (internal capabilities; strategic perspective; learning; growth); external non-financial outcomes (customer orientation)	Stakeholder theory, Transaction cost economics theory	Quantitative	489 owner & executives (150 family firms) (cross-sectional)	S M	Pakistan	Family involvement in the company (through family commitment, continuity, control, enrichment) has a positive influence on the sustainable survival of the company (financial strength, customer orientation, internal capabilities, strategic perspective, learning & growth). This effect is partially mediated by CSR
Antheaume et al. (2013)	Family values; community embeddedness	Internal non-financial outcomes (longevity)	Grounded theory	Qualitative	17 family firms (6 interviews)	M L	France	Longevity of family firms is promoted by interdependencies and the networking of different areas, the embedding of the family in the business as well as the embedding of the business in society and the passing on of capital to the next generation. Sustainable development is preferred to short-term profits
Biscotti et al. (2018)	Family ownership; environmental management systems (knowledge management)	Internal non-financial outcomes (environmental product innovation)	Social identity theory, Institutional theory	Qualitative	262 firms (10 year panel)	–	International	The EMAS-certified environmental management system has a moderating effect in family businesses on the knowledge management practices of employee training and development and, through this, on green product innovation. The ISO 14001 certified environmental management system does not lead to proactive behavior in family and non-family businesses
Choi et al. (2019)	Family ownership; being a chaebol firm (moderator)	Firm performance	–	Quantitative	198 (6 year panel)	L	South Korea	Family ownership has a negative effect on corporate social performance (CSP). This effect is lower (moderation) in chaebol (family-run or controlled conglomerate) firms than in non-chaebol firms
Craig and Dibrell (2006)	Family ownership and management	Financial performance (firm performance); internal non-financial outcomes (firm innovation)	Stewardship theory	Quantitative	396 firms (cross-sectional)	S M	USA	Family firms are better able to promote environmentally friendly policies than non-family firms, although non-family businesses place more emphasis on this. In family businesses, this leads to improved business innovation and higher financial performance
Dangelico (2017)	Family ownership and management	Green product development (differentiation); radicalness or market performance of green products (not significant)	–	Quantitative	188 firms (cross-sectional)	S M L	Italy	Family firms show a positive effect on the differentiation of green product developments. They show no significance in the radicality or market performance of green product
Doluca et al. (2018)	Family ownership and management (no significant difference (but stabilizing effect over time)	Financial outcomes (stabilizing, economic performance); internal non-financial outcomes (stabilizing, environmental product/process innovation)	Stakeholder theory, Institutional theory	Quantitative	633 firm observations (4 year panel)	S M L	Germany	Compared to non-family firms, family firm show fewer environment-related activities and fewer beneficial product, process and organizational innovations and services at the beginning of the study. In the process of the study, a convergence process can be identified, as family businesses are catching up with non-family firms. Family firms also show less volatility in their environmental behavior
Kashmiri and Mahajan (2010)	Family name	Financial outcomes; return on assets (performance)	–	Quantitative	130 family firms (5 year panel)	L	USA	Family firms that carry the family name compared to family businesses without the family name have higher corporate citizenship, representation of their customers' voice on the top management team, higher strategic focus (resource allocation to advertising), and better performance (higher return on investment). Performance is mediated by higher corporate citizenship and stronger strategic focus
Kashmiri and Mahajan (2014a)	Family management; family name; ethical product-related behavior (mediator)	External non-financial outcomes; financial outcomes (increasing of the abnormal stock returns)	–	Quantitative	107 family firms & 1294 product introduction announcements (3 year panel)	L	USA	Family firms that bear the founder’s name have higher abnormal stock returns surrounding the firm's new product introductions compared to family firms without the founder's name. This effect is mediated by the ethical behavior of the firms, including the fact that they are less involved in product-related controversies
Kashmiri and Mahajan (2014b)	Family ownership and management (combined)	Firm performance	–	Quantitative	275 firms (10 year panel)	L	USA	CSR mediates the relationship between family ownership/management and firm performance. In contrast to non-family firms, family firms do not reduce their CSR during a recession, which results in higher firm performance
Liu et al. (2017)	Family ownership and family management (combined)	Accrual-based earnings management; real earnings management	–	Quantitative	(8 year panel)	L	USA	Family firms tend to have a higher CSR performance than non-family firms and have less accrual-based earnings management and real earnings management. Furthermore, the positive effect of CSR on earnings management described in the research is attributable to family involvement
Niehm et al. (2008)	Family firm size	Subjective business performance; objective financial performance	–	Quantitative	221 family-firm households (2 year panel)	S	USA	The size of a family firm has a positive impact on the CSR dimension “community support,” which in turn has a positive effect on the objective (financial) performance of the firm. In contrast, the CSR dimension “commitment to the community” is positively linked to forms’ subjective performance
O’Boyle et al. (2010)	Family involvement	Financial outcomes (financial performance)	–	Quantitative	526 family firms (cross-sectional)	S M	USA	The ethical focus of a company mediates the relationship between family involvement and financial results. The factors value congruence and participative continuance have a positive effect on ethical focus, while ownership and control and professionalism show a negative effect
Singal (2014)	Family ownership and management (combined)	Financial performance	Instrumental theory	Quantitative	534 firms (11 year panel)	–	USA	Family firms have a better financial and CSR performance than non-family firms, whereby the higher CSR performance in family firms is triggered by their better financial performance. Family firms continue to invest more in mitigating concerns than in positive initiatives to build strengths in CSR performance. Family firms’ higher CSR inclination further has a positive impact on their future financial performance
Wu et al. (2014)	Family ownership	Costs of capital	–	Quantitative	482 firms (4 year panel)	–	Taiwan	Firms with CSR awards have lower cost of capital. Family firms with CSR have lower cost of capital than do non-family firms with CSR
Zientara (2017)	SEW	Family firm reputation	SEW	Theoretical	–	–	–	Due to SEW orientation, family firms use an instrumental and selective CSR approach rather than a holistic or normative one. This often leads to socially responsible behavior towards external stakeholders, but irresponsible behavior towards internal stakeholders. Family firms pick those CSR initiatives that serve their own interests, like improving the firm’s image and reputation

*S* small, *M* medium, *L* large (listed)
